# Supplementary material for: Local incomplete combustion emissions define the PM2.5 oxidative potential in Northern India
Source: Nat Commun. 2024 Apr 25;15:3517. doi: 10.1038/s41467-024-47785-5 (PMC11045729; doi:10.1038/s41467-024-47785-5)
Supplement: Supplementary file 1 — Supplementary Information [file 41467_2024_47785_MOESM1_ESM.pdf]

Supplementary Information

Local incomplete combustion emissions define the $PM_{2.5}$ oxidative potential in Northern India

Deepika Bhattu*, Sachchida Nand Tripathi*, Himadri Sekhar Bhowmik, Vaios Moschos, Chuan Ping Lee, Martin Rauber, Gary Salazar, Gülcin Abbaszade, Tianqu Cui, Jay G. Slowik, Pawan Vats, Suneeti Mishra, Vipul Lalchandani, Rangu Satish, Pragati Rai, Roberto Casotto, Anna Tobler, Varun Kumar, Yufang Hao, Lu Qi, Peeyush Khare, Manousos Ioannis Manousakas, Qiyuan Wang, Yuemei Han, Jie Tian, Sophie Darfeuil, Mari Cruz Minguillon, Christoph Hueglin, Sébastien Conil, Neeraj Rastogi, Atul Kumar Srivastava, Dilip Ganguly, Sasa Bjelic, Francesco Canonaco, Jürgen Schnelle-Kreis, Pamela A. Dominutti, Jean-Luc Jaffrezo, Sönke Szidat, Yang Chen, Junji Cao, Urs Baltensperger, Gaëlle Uzu, Kaspar R. Daellenbach, Imad El Haddad*, André S. H. Prévôt*

*Corresponding author. Email: dbhattu@iitj.ac.in (D.B.); snt@iitk.ac.in (S.N.T.); imad.el-haddad@psi.ch (I.E.H.); andre.prevot@psi.ch (A. S. H. P.)

This PDF file includes:

1. Supplementary Methods (Method 1-5)
2. Supplementary Figures (Figure 1-12)
3. Supplementary Tables (Table 1-8)
4. Supplementary References

Supplementary Method 1: Sampling site details

New Delhi is situated 160 km south of the Himalayas and near the Thar Desert. It is surrounded by the adjoining state of Haryana on three sides (North, West and South) and Uttar Pradesh on the other side. During winter, New Delhi experiences prevailing northwesterly winds originating in the Himalayas and crossing northern plain whereas during summer, south-westerly winds prevail (Supplementary Fig. 9). To understand the effect of local sources and regional transport of the pollutants in the capital city, 5 sites were selected. Two sites were in Delhi (North-west: Indian Institute of Tropical Meteorology Delhi (IITMD) representative of urban background, South-west: Indian Institute of Technology Delhi (IITD) as urban roadside). Two sites encompassing Delhi were in the north-west direction (Chaudhary Charan Singh Haryana Agricultural University (CCSHAU) Hisar, Haryana) and south-east direction (Manav Rachna International University, Faridabad (MRIUF)) as upwind rural background site and a sub-urban industrial site, respectively. The last downwind site in south-east direction is a sub-urban Kanpur site (Indian Institute of Technology Kanpur (IITK).

The Delhi urban background site is in central urban part of Delhi, surrounded by forest areas and residential colonies whereas Delhi urban roadside site is an urban residential site surrounded by roads with heavy traffic (<100 m away) and industries located in the north-west and east direction. Further, sub-urban industrial site is a residential campus near the main road with both heavy- and light-duty vehicles and surrounded by industries as well as sewage treatment plants in the north direction. The north-west rural background upwind site is an Agromet Observatory situated in the agricultural research farm at CCSHAU, Hisar.

The Delhi urban roadside site experiences high wind speeds and less variable RH conditions as compared to the urban background and sub-urban industrial site. The next sub-urban sampling site (IITK) is ~600 km to the South-East. It is also a residential campus in proximity of roads with moderate traffic (~800 m) and upwind of a coal power plant (Panki). More details on the sampling sites are provided in Bhowmik et al.[1] and Tripathi et al.[2].

Supplementary Method 2: Filter collection, storage, and transportation

High volume $PM_{2.5}$ samplers (Thermo at the Delhi urban roadside site and Tisch at the other 4 sites with a flowrate of 1.13 m^3 min^{-1}) were used to collect 893 samples (865 samples and 28 field blanks) from January to May 2018 on quartz fiber filters (8" x 10", pre-combusted (~350 °C) Pallflex® at Delhi urban roadside site and Whatman QMA at the other 4 sites). The samplers were installed at the roof top of the Agromet Observatory, Department of Agricultural Meteorology, CCSHAU (29.10◦N, 75.46◦E; ~215 m amsl) Hisar (~5 m above ground), main building of IITM Delhi (~15 m above ground; 28.63◦N, 77.167◦E; ~220 m amsl), Centre for Atmospheric Science Building (~15 m above ground) at IIT Delhi (28.54◦N, 77.19◦E; ~218 m amsl), C Block of MRIUF (~7 m above ground; 28.45◦N, 77.28◦E ~278 m amsl) and Centre for Environmental Science and Engineering (CESE), IIT Kanpur (~8 m above ground; 26.51◦N, 80.23◦E ~142 m amsl). Filters were wrapped in aluminium foils, sealed in a zipped plastic bag and stored at -18 to -20 °C at all sampling sites. Filters were shipped to IIT Kanpur once every two months in a thermally insulated box filled with dry ice to avoid loss of semi-volatile species. These filters were again stored at -20 °C on receipt and later kept at room temperature (20 °C) for 30 minutes before cutting the whole filter area into two halves. This procedure was performed in a temperature and RH-controlled clean room and one half of all the filters was wrapped in a new aluminium foil, sealed in a zipped plastic bag, and packed with dry ice for shipment to PSI, Switzerland. A total of 330 filter samples (including field blanks and repeats), referred to as "2018 filters" in the following text, were selected from: (1) every 6th sampling day (day and night filters until mid-March and 1 filter from every third day for the rest of the period), and (2) additional periods of interest identified for analysis, along with the days immediately prior and posterior to such periods. $PM_{2.5}$ mass was reconstructed based on all the measured components (organics, inorganic ions, and elements as their oxides).

Supplementary Method 3: WSOC measurement vs estimation

We compared TOC analyzer-measured WSOC and WSOC derived from AMS [i.e., total AMS-OA/ $(OM/OC)_{bulk}$*(^{15}NO + $^{15}NO_2$)], where (^{15}NO + $^{15}NO_2$) was used to correct for any changes in the AMS signal intensity. It was found that the periods with high concentrations of dominant cations (Ca^{2+}, Mg^{2+} and K^+) have undergone recombination with labelled ions, yielding compounds that are not readily vaporized, thus resulting in negative bias for the AMS-derived WSOC.

Supplementary Method 4: Aerosol instrumentation and measurements

A) AMS and EESI-ToF measurements: The AMS was set up to provide average (40 seconds) mass spectra in the mass to charge (*m/z*) range of 12- 440 (V-mode). The acquisition rate for the EESI mass spectra for up to *m/z* 960 was 1 Hz. Overall, a total of 394 samples (including 28 field

blanks, 36 sample repeats and 12 pure labelled and 13 unlabeled $NH_4{}^{15}NO_3$ and $(NH_4)_2{}^{34}SO_4$ standards) were measured. The details of AMS operating principles, calibration procedures, and procedures for data analysis are provided elsewhere[3], as well as the detailed description of the EESI-ToF and its operating principles[4]. Briefly, the EESI used a 200 ppm NaI solution consisting of a 1:1 water to acetonitrile mixture as electrospray solution (ES) and was configured for positive ion detection. The aerosol stream from the nebulizer intersected a jet spray of charged droplets generated from an untreated fused silica capillary (360 µm outer diameter and 50 µm inner diameter) (BGB Analytik, AG) contained in an electric field. The aerosol components soluble in the electrospray jet of charged droplets were extracted, ionized, and ejected into the gas phase for detection by the LToF-MS. A potential difference of 3.0 kV relative to the MS interface was applied to the ES solution and an air pressure difference of 500 mbar was used to drive the ES solution through the capillary to make ES droplets. The stability and performance of both instruments were tracked by nebulizing NH_4NO_3 and $(NH_4)_2SO_4$ standard solutions. Due to negligible thermal degradation and ion-induced fragmentation[4,5], near-molecular level (i.e., molecular ion formulae) OA composition was obtained. For AMS data analysis, Squirrel v1.59B was used for *m/z* calibration and baseline subtraction, and PIKA (Peak Integration by Key Analysis) v1.19B for high resolution mass spectrum analysis[6] in the IGOR Pro software package 6.37. A total of 785 fragments and the isotope ions up to *m/z* 182 were fitted in HR peak fitting procedure. EESI data processing was performed using Tofware version 2.5.7 (Tofwerk AG, Switzerland). Ten seconds pre-averaged data was used for high-resolution peak fitting. A total of 2003 ions were fitted in the selected mass transmission window between *m/z* 120 and 444.

B) Radiocarbon measurements: The ^{14}C content of TC was measured using a one-step combustion protocol under pure O_2 (99.9995 %) at 760 °C for 400 s[7] using an elemental analyzer coupled with the accelerator mass spectrometer Mini Carbon Dating System (MICADAS) at the Laboratory for the Analysis of Radiocarbon (LARA; University of Bern, Switzerland)[8,9]. The EC fraction was separated by a thermo-optical OC/EC analyzer (Model 5L, Sunset Laboratory, USA) coupled online with the MICADAS[10].

For the determination of ^{14}C of EC, the Swiss_4S protocol[8] had been developed for samples from Switzerland or other samples with similar characteristics/mass loading. However, preliminary analysis with water extraction showed that OC in the Indian PM samples was less water-soluble and more methanol-soluble than the Swiss samples. Therefore, the temperatures applied in the Swiss protocol for OC removal resulted in high charring (i.e., a large fraction of OC got converted to EC, eventually leading to artificially high EC yields, while a large fraction of the EC was lost in this OC removal step. Therefore, a modified extraction and desorption temperature protocol was developed (Bern-India_4S) to achieve efficient EC separation for these samples: we used methanol instead of water extraction for OC removal with the Sunset OC/EC analyzer and reduced the temperature in step 2 from 475 °C to 425 °C. This led to efficient removal with minimized charring and acceptable EC yields.

The ^{14}C results are expressed as fractions of modern (f_M), i.e., the ratio of the $^{14}C/^{12}C$ content of the sample related to the isotope ratio of the reference year 1950[11]. The data analysis was carried out accounting for the blank correction (one field blank per site was analyzed, not relevant for EC), decay of ^{14}C since the 1950s, nuclear bomb correction, OC charring (average and 1 standard deviation: 4 ± 3%; lower at the urban roadside), and EC yield after OC removal (average and 1 standard deviation: 72 ± 9%; no significant site-to-site variability)[12,13].

Non-fossil fractions (f_{NF}) were determined from their corresponding f_M values and reference values for pure non-fossil sources ($f_{NF,ref}$) by $f_{NF} = f_M / f_{NF,ref}$. For the bomb-peak correction[12], based on the AMS-PMF results we considered that biomass-burning (bb) and biogenic (bio) sources contribute 90% and 10%, respectively, to the $f_{NF,ref}$; $f_{M,bb}$ and $f_{M,bio}$ values from Zotter et al.[13]. Based on our tests, we used the lower value of 0.07 for the EC yield slope from Zotter et al.[13] to correct the f_M values for charring and EC losses. The fraction of fossil-fuel sources was calculated by $f_{FF} = 1 - f_{NF}$. The apportionment to fossil and non-fossil OC was based on mass balance calculations (OC = TC – EC) (Supplementary Fig. 10). The uncertainties were determined by error propagation. The mass concentration uncertainties were assumed to be 10% for EC and 6% for OC and TC (ref.[12], typical values for EUSAAR2).

C) Organic Carbon- Elemental Carbon (OC-EC): Organic and elemental carbon was measured by a Sunset EC-OC analyzer (Model 4F, Sunset Laboratory Inc. USA) using the EUSAAR-2 thermal-optical transmittance protocol[14]. Details of the analysis method and choice of protocol are described elsewhere[1].

D) Water-soluble OC (WSOC), water soluble inorganic carbon (WSIC) and ions: Water-soluble organic carbon was measured by a total organic carbon analyzer (Model: Shimadzu-TOC-L-CPN, Shimadzu Corporation) by catalytically oxidizing water-soluble total carbon and measuring the resultant CO_2 using a non-dispersive infrared (NDIR) detector[1]. WSIC was measured from CO_2 evolved after acidification of water extracts. The concentrations of major ions (K^+, Na^+, NH_4^+, Cl^-, NO_3^-, SO_4^{2-}) were measured by ion chromatography[1].

E) Trace elements: 29 trace elements (Li, Mg, As, Ca, Sc, Ti, V, Cr, Mn, Fe, Co, Ni, Cu, Se, Rb, Sr, Zr, Mo, Pd, Cd, ln, Sn, Sb, Cs, Ba, Ce, Pt, Tl and Pb) were measured by inductively coupled plasma mass spectrometry (ICP-MS; Thermo Scientific iCAP Q ICP-MS). Briefly, filter samples were digested by an acid mix of HF, HNO_3 and $HClO_4$ solution. Percentage recovery was calculated compared with high purity multi-element (35 trace elements) standards (soluble in 1% HNO_3, 100 ppm) (Sigma Aldrich)[15].

F) Targeted organic compounds: A range of 81 targeted organic compounds including 15 dicarboxylic acids, 22 polycyclic aromatic hydrocarbons (PAHs), 8 oxygenated polycyclic aromatic hydrocarbons (oxy-PAHs), 3 anhydrous sugars, 2 resin acids, 2 alkaloids, 10 hopanes, 10 n-alkanes, 7 higher n-alkanes and 2 lignin pyrolysis products were determined to support the source apportionment results. A total of 140 samples were measured from the CCSHAU, IITMD, IITD and MRIUF sites. Details of the methods are described in ref.[16,17].

G) $PM_{2.5}$ oxidative potential: A proxy for health-relevant exposure of $PM_{2.5}$ through oxidative damages was assessed by determining the oxidative potential of PM collected on filters, using three different acellular assays (dithiothreitol: DTT; 2´,7´-dichlororfluorescin: DCFH and ascorbic acid: AA). Details of the methods are described elsewhere[18,19]. Briefly, all PM samples, including field blanks, were extracted at iso-concentration (25 µg ml^{-1}) using simulated lung fluid (Gamble solution + dipalmitoylphosphatidylcholine (DPPC) solution) and further subjected to different assays in triplicate, without filtering the extracts to keep the PM in contact with the lung antioxidant or surrogate. Multi-assay measurements were performed to obtain a comprehensive picture of the effects of different reactive oxygen species (ROS) generation mechanisms: particle-bound ROS (DCFH) and catalytic generation via redox-active constituents (AA and DTT). The DTT assay method measures the electron transfer ability of PM, thereby producing ROS. It mimics interaction of surrogates (nicotinamide adenine dinucleotide: NADH and nicotinamide adenine dinucleotide phosphate-oxidase: NADPH) with PM[18] and is sensitive to both soluble metals and organic carbon (HULIS, quinones and water-soluble organics)[20], and, as a thiol-based reagent, provides a balanced answer to a wide range of pollutants. The AA assay is a simplified version of the respiratory tract lining fluid (RTLF) assay which also measures the electron-transfer ability of PM from AA to O_2[18]. It is sensitive to redox-active transition metals (Fe and Cu) and mainly specific organic aerosol sources (biomass burning). DCFH, on the other hand, is a fluorescent probe sensitive to peroxides (H_2O_2 and organic peroxides). It measures particle-bound ROS but is also known to be sensitive to reactive nitrogen species[21].

Supplementary Method 5: Data analysis

A) **2D hierarchical clustering on AMS-derived factor profiles:** For each AMS-derived factor, a specific cluster of closely associated ions was observed and is discussed below.

CPOA was identified by the relatively high abundance of CHN ions signals (*m/z* 95 {$C_5H_7N_2$}, *m/z* 96 {$C_5H_8N_2$}, *m/z* 108 {$C_6H_8N_2$}, *m/z* 109 {$C_6H_9N_2$}, *m/z* 110 {$C_6H_{10}N_2$}, *m/z* 122 {$C_8H_{12}N$}, and *m/z* 123 {$C_8H_{13}N$}) and contributions by some hydrocarbons (HC) and CHO ions ($C_xH_yO_1$ and $C_xH_yO_3$), and a few CHNO ions.

BBOA had high abundance of *m/z* 60 {$C_2H_4O_2$} and *m/z* 73 {$C_3H_5O_2$}. It correlated (Pearson's r=0.75; n=140) with levoglucosan measured by GC-MS. The signal intensity of these representative ions was 10 times higher than other unique and abundant ions like *m/z* 126 {$C_7H_{10}O_2$}, *m/z* 73 {$C_2H_3NO_2$}, *m/z* 61 {$C_2H_5O_2$}, *m/z* 60 {CH_2NO_2} and *m/z* 57 {C_3H_5O}.

COOA was identified as a highly oxidized factor with *m/z* 28 (CO^+) and *m/z* 44 (CO_2^+) as representative ions.

UOOA was identified by *m/z* 43 {C_2H_3O} and other unique ions with formula $C_xH_yO_1$ and $C_xH_yO_2$, including *m/z* 58 {C_3H_6O}, *m/z* 71 {C_4H_7O}, *m/z* 85 {$C_4H_5O_2$}, *m/z* 83 {C_5H_7O}, *m/z* 95 {C_6H_7O}, *m/z* 59 {C_3H_7O}, *m/z* 99 {$C_5H_7O_2$}, *m/z* 97 {C_6H_9O}, *m/z* 111 {$C_6H_7O_2$}.

B) HOA estimation: HOA estimation was performed using the fossil and non-fossil fractions of EC and levoglucosan by employing two different approaches.

a) In approach 1, the following steps were used:

i) Correlation of EC_{nf} (*y*-axis) and levoglucosan (*x*-axis): A moderate correlation (Pearson's r = 0.7) was obtained (n=44; selected samples from upwind rural background, Delhi urban background and urban roadside) with a slope of 1.08 ± 0.17 and an intercept of 0.6 ± 0.2. $EC_{nf,estimated}$ was calculated using the slope of 1.08 for the remaining 140 samples from the same sites that had the levoglucosan concentration measured. Later, $EC_{f,estimated}$ was estimated using Eq. S1 as follows.

$$EC_{f,estimated} = Sunset\ EC_{measured} - EC_{nf,estimated} \qquad \textbf{Eq. S1}$$

Further, uncertainties ($\sigma_{EC_{nf,estimated}}$ & $\sigma_{EC_{f,estimated}}$) were estimated using Eq. S2 and Eq. S4.

$$\sigma_{EC_{nf,estimated}} = \sqrt[2]{\left(\frac{\sigma_{slope}}{slope}\right)^2 + \left(\frac{\sigma_{levo}}{C_{levo}}\right)^2} \qquad \textbf{Eq. S2}$$

$$\sigma_{levoglucosan} = \left\{\frac{5}{6} * MDL_{levo},\ \text{if } C_{levo} < MDL_{levo}\right. \qquad \textbf{Eq. S3}$$ [22,23]

$$\text{Otherwise, } \left\{\sqrt{(U_{levo} * C_{levo})^2 + (MDL_{levo})^2}\right.$$

where MDL_{levo} is the minimum detection limit of the instrument for levoglucosan (0.03 ng m^{-3}) and U_{levo} is the analytical uncertainty (10%).

$$\sigma_{EC_{f,estimated}} = \sqrt[2]{\sigma_{EC_{nf,estimated}}{}^2 + \sigma_{EC}{}^2} \qquad \textbf{Eq. S4}$$

ii) We obtained a slope (1.5 ± 0.1; n=38; Pearson's r = 0.8) of *HOC* vs. EC_f, with EC_f derived from radiocarbon (^{14}C) analysis and HOC obtained as follows

$$HOC = \frac{HOA_{AMS_{Delhi\ urban\ roadside}/ACSM_{Delhi\ urban\ background}}}{\left(\frac{OM}{OC}=1.25\right)_{HOA}} \qquad \textbf{Eq. S5}$$

iii) $HOC_{estimated}$ for the remaining 140 samples was obtained by multiplying $EC_{f,estimated}$ computed in step (i) with the slope of 1.5 obtained in step (ii) (Eq.6). $HOA_{estimated}$ was further computed using the OM:OC ratio of 1.25 again and uncertainty was estimated using Eq. S7.

$$HOC_{estimated} = 1.5 * EC_{f,estimated} \qquad \textbf{Eq. S6}$$

$$\sigma_{HOA_{estimated}} = \sqrt[2]{\sigma_{EC_{f,estimated}}{}^2 + \left(\frac{\sigma_{slope\ (HOC\ vs\ EC_f)}}{slope\ (HOC\ vs\ EC_f)}\right)^2} \qquad \textbf{Eq. S7}$$

In approach 2, the following steps were used:

i) The average EC_f/EC_{nf} ratio of 2.4 ± 0.8 (at upwind rural background and Delhi urban sites) was calculated

$$EC = EC_f + EC_{nf} = 2.4 * EC_{nf} + EC_{nf} = 3.4 * EC_{nf} \qquad \textbf{Eq. S8}$$

$$EC_{nf} = 0.3 * EC \qquad \textbf{Eq. S9}$$

ii) $EC_{nf,estimated}$ was computed for the remaining sites/samples using a factor of 0.3 times the total Sunset-EC from Eq. S9.

iii) $EC_{f,estimated}$ (*y*-axis) for all sites by subtracting $EC_{nf,estimated}$ from total Sunset-EC and compared with $HOC_{estimated}$ (*x*-axis) obtained from approach 1. A slope of 0.6 was obtained which is further used to calculate $HOC_{estimated}$ for downwind Kanpur site outside Delhi, where only total *EC* was available.

Finally, approach 1 and 2 were compared to evaluate their compatibility to estimate HOA for the sites where either EC_f (sub-urban industrial site in Delhi and downwind Kanpur site outside Delhi) or levoglucosan concentrations were not available. However, an R^2 of 0.96 and slope of 0.98 while comparing estimated HOA suggested the suitability of approach 2 in the absence of specific source markers i.e., levoglucosan.

C) Multi-linear regression for fossil and non-fossil fractions of OA sources

The uncertainty-weighted mass concentration time series (i) of species-specific recovery corrected factors obtained after AMS-PMF were used in Eq. S10. BBOC was assumed to be completely non-fossil.

$$OC_{nf,i} - BBOC_i = a * CPOC_i + c * COOC_i + d * UOOC_i \qquad \textbf{Eq. S10}$$

Where a, b, c and d are the fitting coefficients. The fossil fraction of each factor was computed by subtracting their respective non-fossil fraction from 1. These values represent an average over both the cold and warm period which might have a certain seasonality. The uncertainties were estimated by performing 1000 bootstrap runs. A similar approach was used to calculate the fossil fractions of the factors by replacing OC_{nf} with OC_f- HOC_{est} (Eq. S11), based on the assumption that HOC_{est} is completely fossil and assumed to have similar relative ionization efficiency (RIE) as other fractions of OC.

$$OC_{f,i} - HOC_{est,i} = a * CPOC_i + b * BBOC_i + c * COOC_i + d * UOOC_i \quad \textbf{Eq. S11}$$

The results from multilinear regression on both the fossil and non-fossil fractions were similar, as shown in Supplementary Table 6.

D) Sources of trace elements

Due to on-site contamination, only six (Mn, Cu, Cd, Sn, Sb, and Pb) of the measured elements had sample concentrations higher than the threshold (field blank average + 3 standard deviations). The spatial and temporal variation, and relative contribution of these selected trace elements with two water soluble marker ions (Na^+ and K^+) is shown in Supplementary Fig. 11. The Pearson's r between water-soluble K^+ ions (IC) and total K from acid-digested samples (ICP-MS) is 0.7. The values for the acid-digested samples are nearly twice those of the water-soluble ones.

To determine the sources of the trace elements, an unconstrained PMF was performed for 3-14 factors based on elements including Na^+, K^+, Li, Mg, Ca, Ti, V, Cr, Mn, Fe, Co, Ni, Cu, Se, Sr, Zr, Mo, Pd, Cd, Sn, Sb, Cs, Ba, La, Ce, Pt, and Pb from all five sites. We used all measured elements in the PMF matrix to estimate the contribution of elements in the identified contamination factors. The data matrix (j=294 samples) included the mass concentrations of the mentioned elements and the error matrix was computed by using Eq. 3 and 4[22,23]. The field blanks were treated as real samples and included with the aerosol samples in the data and corresponding error matrix. MDL is the element-specific minimum detection limit of instrument and p is the analytical uncertainty (3%). Two random seed runs were performed for each factor solution making a total of 24 runs. Supplementary Fig. 12 shows the optimum base-case solution chosen after investigating the preliminary diagnostics. The optimum solution with the lowest Q/Q_{exp} was observed for the 8-factor solution, after which the relative change ($\Delta Q/Q_{\text{exp}}$) is small (<0.25). Three factors identified as K^+-Na^+ rich, Cu-Cd rich, and Pb-Sn-Sb rich and are discussed below, and the remaining five (factors 2a, 2b, 2c, 2d and 2e) were treated as contamination due to their similar contribution in both the field blanks and aerosol samples, as shown in Supplementary Fig. 12.

K^+-Na^+ rich: Na^+- and K^+-dominated factor could potentially be present as a salt when combined with another anion (Cl^-) or inorganic ion (NO_3^- or SO_4^{2-}). At coastal sites (marine aerosols), sea salt is the major source of Na^+ ion, however this is unlikely in the present study as our sampling sites are quite far (1500 km) away from coastal areas. It could be related to multiple anthropogenic sources such as open trash/refuse burning, coal/oil and biomass combustion, and vehicular emissions, as the seasonal K^+ to Na^+ ratios are in the range of 0.5-1.7[24], much higher than that of sea salt (0.036).

Cu-Cd rich: This factor potentially originates from either electrical/electronic equipment waste incineration or burning plastic/open waste[25].

Pb-Sn-Sb rich: This factor has multiple potential sources. It can originate from open waste/plastic burning, lead smelting industries and brake wear (non-tailpipe traffic emissions)[25].

In the 7-factor solution, the Pb-Sn-Sb factor is mixed with Cd, and the K^+-Na^+ rich factor is mixed with Cu, the combination of which in the 8-factor solution results in separate factors that have already been identified as separate sources from electronic and electrical waste incineration and open waste/plastic burning, respectively, at the same site (i.e., IITD) during real-time monitoring and source apportionment of trace elements (offline vs. online PMF (n=35); R^2: 0.76 (Cu-Cd rich) and 0.77 (Pb-Sn-Sb rich)[25]. Further, in the 9-factor solution, a split of the contamination factor 2d was identified. Supplementary Fig. 12 demonstrates the trace element factor profiles from the final 8-factor solution, as well as their concentratons and relative contributions.

E) Stepwise linear regression modeling of $PM_{2.5}$-OP sources

The model adds and removes predictor variables by forward and backward stepwise regression based on the "Akaike information criterion (AIC)" and prevents over-fitting. We have not included any interaction term among predictors and response variables although earlier studies have shown the possibility of both synergistic and antagonistic effects due to organics-metal and metal-metal interaction[26]. In this least-square estimation method, AIC is defined as the sum of twice the number of predictor variables, k, and N times the log of the variance of the noise, as given in Eq. S12.

$$AIC = 2k + N * \log\left(\frac{RSS}{N}\right) \quad \textbf{Eq. S12}$$

Where RSS is the residual sum of squares and N corresponds to the sample size. Model inputs included averaged PMF-derived time series (from BS runs) of OA (CPOA, BBOA, COOA, UOOA and $HOA_{\text{estimated}}$), trace elements (3) sources as well as their contamination factors and measured volume-normalized OP concentrations ($DCFH_v$, DTT_v, and AA_v). The linear least-square solver "lsqlin" was used to put non-negative constraints on the predictor variables in order to obtain the final values of the OP strength (OP_m, nmol μg^{-1}) and the contribution of the OA and trace elements sources (shown as cumulative density function (CDF) plots in Supplementary Fig. 4a). We observed that unconstrained fitting does not affect the coefficient of predictor variables corresponding to the ambient factors.

Supplementary Figures

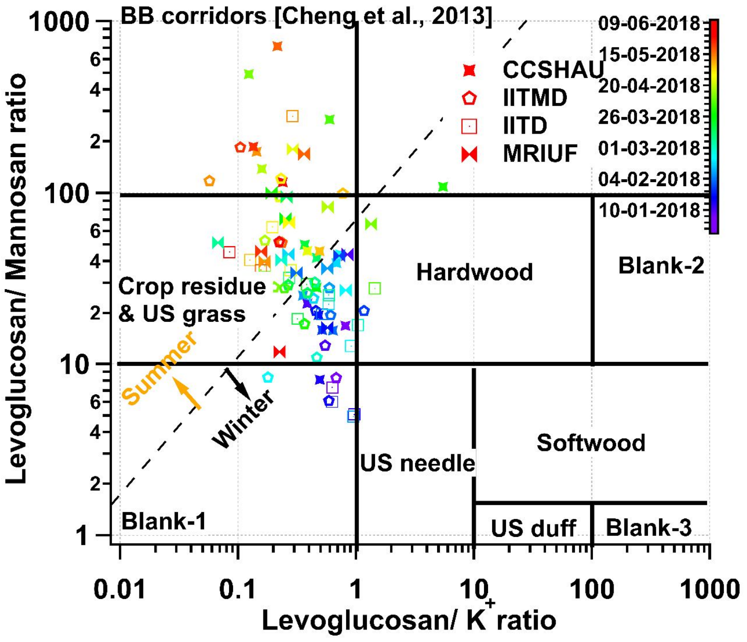

Supplementary Fig. 1: Biomass burning corridors *(27)*. Levoglucosan to mannosan ratio vs. levoglucosan to K^+ ratio to investigate the molecular diversity in biomass burning and spatiotemporally distinguish the change in the type of biofuels combusted.

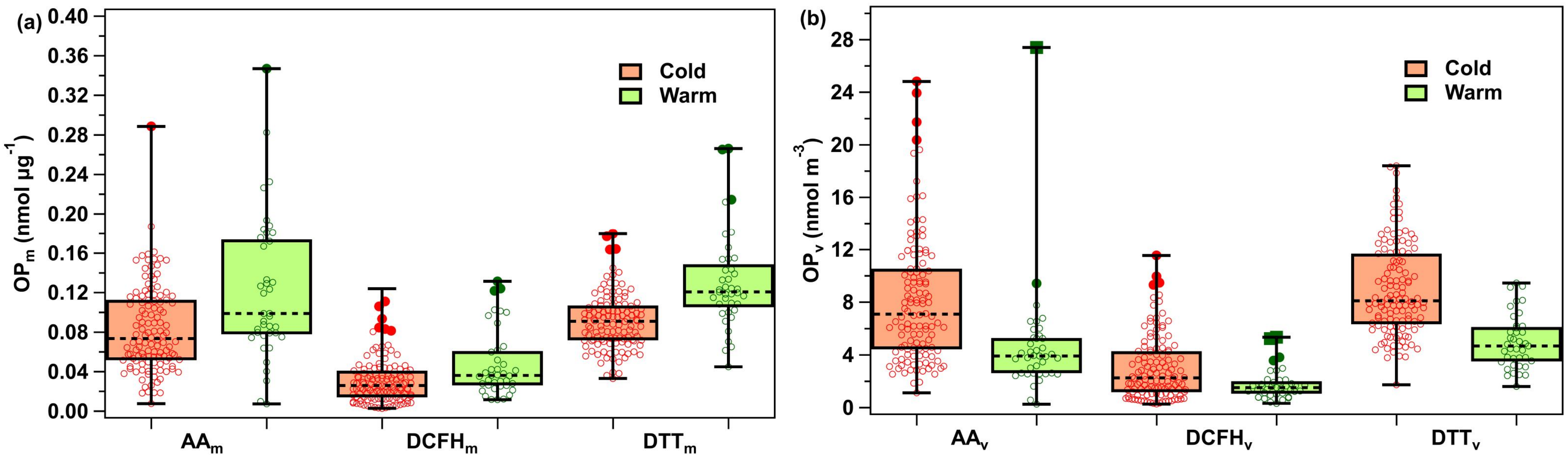

Supplementary Fig. 2: Seasonal variation of (a) mass- (OP$_m$, per µg of $PM_{2.5}$) and (b) volume-normalized (OP$_v$, per m^3 of air) OP values for all 3 assays (DCFH, DTT and AA) for cold and warm period. Box whisker plots [line/box: median and 25th-75th percentile; upper and lower end of whisker: 5th-95th percentile] are prepared from spatial- and temporal-values.

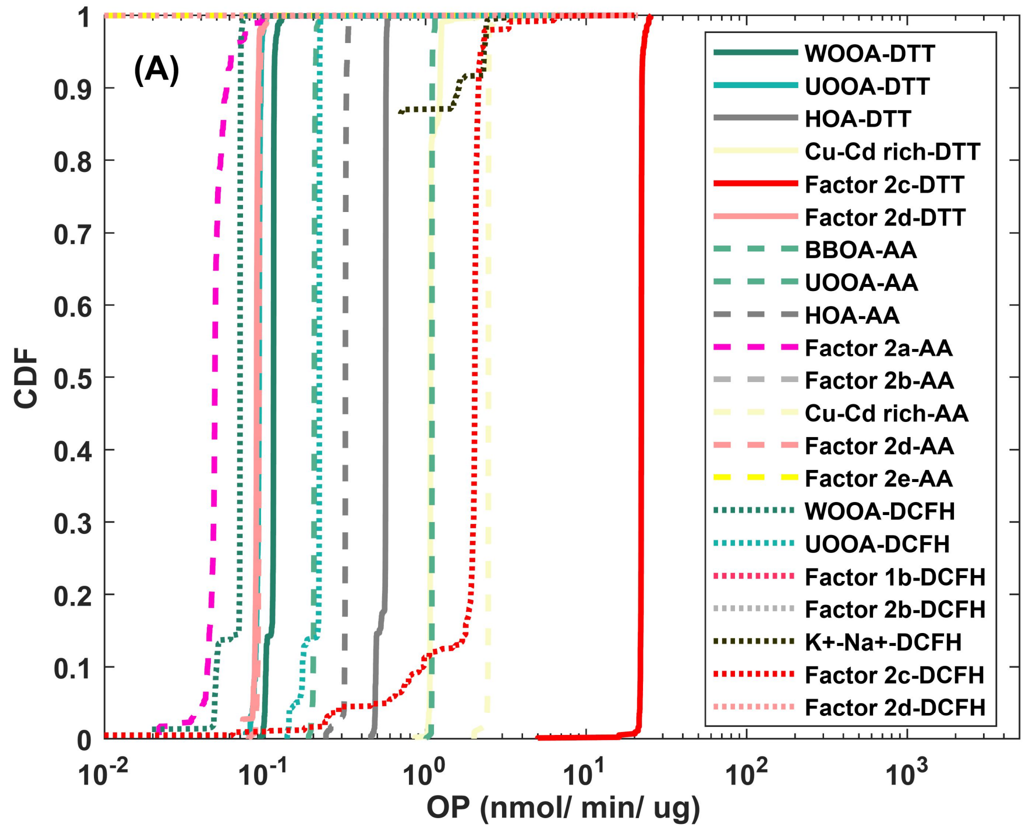

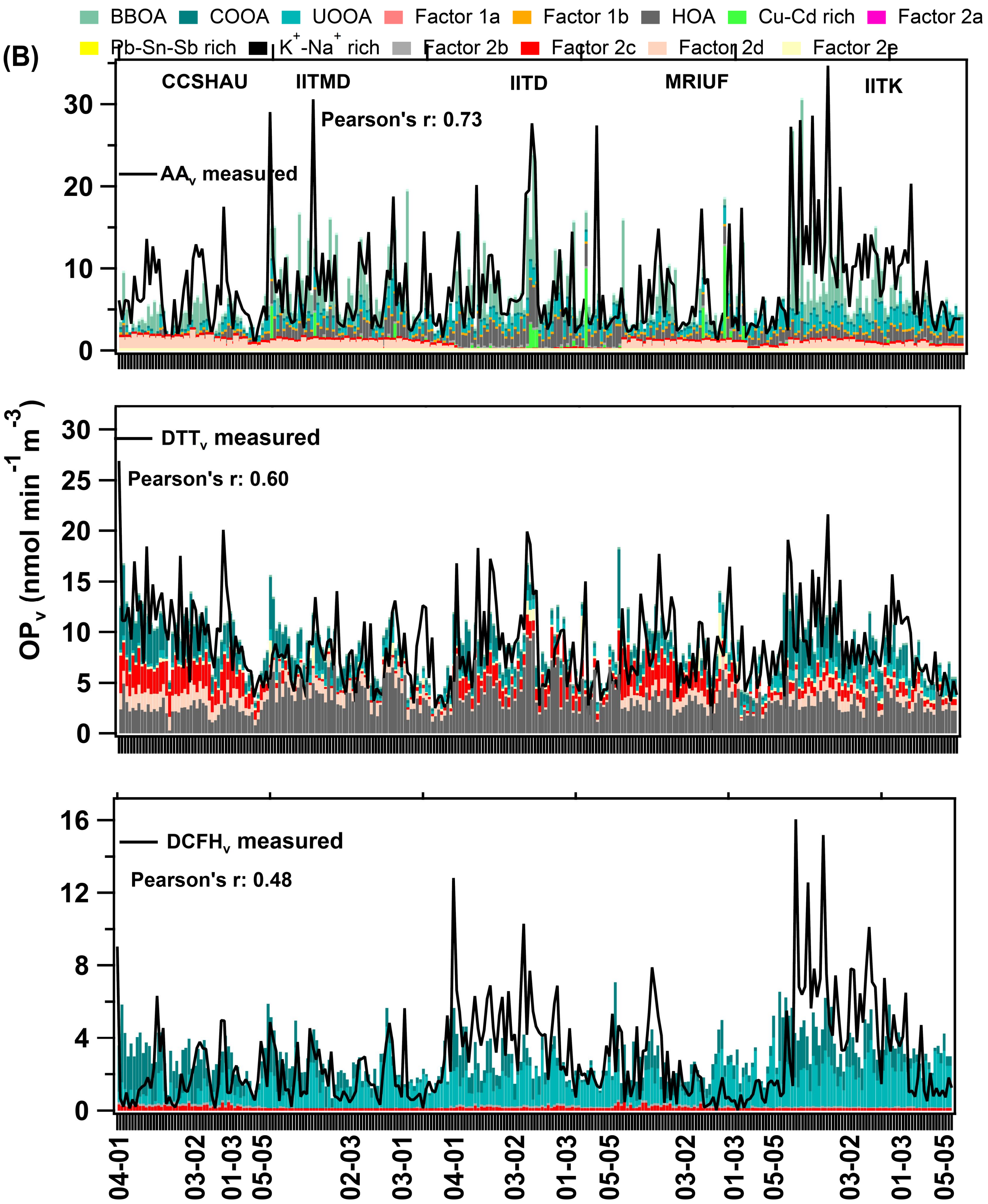

Supplementary Fig. 3: OP_m for OA and elemental sources, and comparison of measured vs. modelled OP_v. (a) OP_m (OP per unit aerosol mass; nmol min^{-1} μg^{-1}) for different OA and elemental sources (displayed as cumulative density functions, CDF) obtained from multi-linear regression (1000 bootstrap runs) between measured OP_v (as predictor variable), and OA and elemental source concentrations (as response variable) and their contamination sources. OA sources consist of COOA, UOOA, BBOA, CPOA, and HOA_{est}, and elemental sources of Cu-Cd rich, Pb-Sn-Sb rich, and K^+-Na^+ rich profiles. Factors 1a and 1b are the contamination factors in OA-PMF and Factors 2a-2e in elemental PMF. **(b)** Comparison of source-specific stacked OP_v (as the product of real source mass contribution after subtracting contamination sources and OP_m) and total measured OP_v for AA, DCFH and DTT.

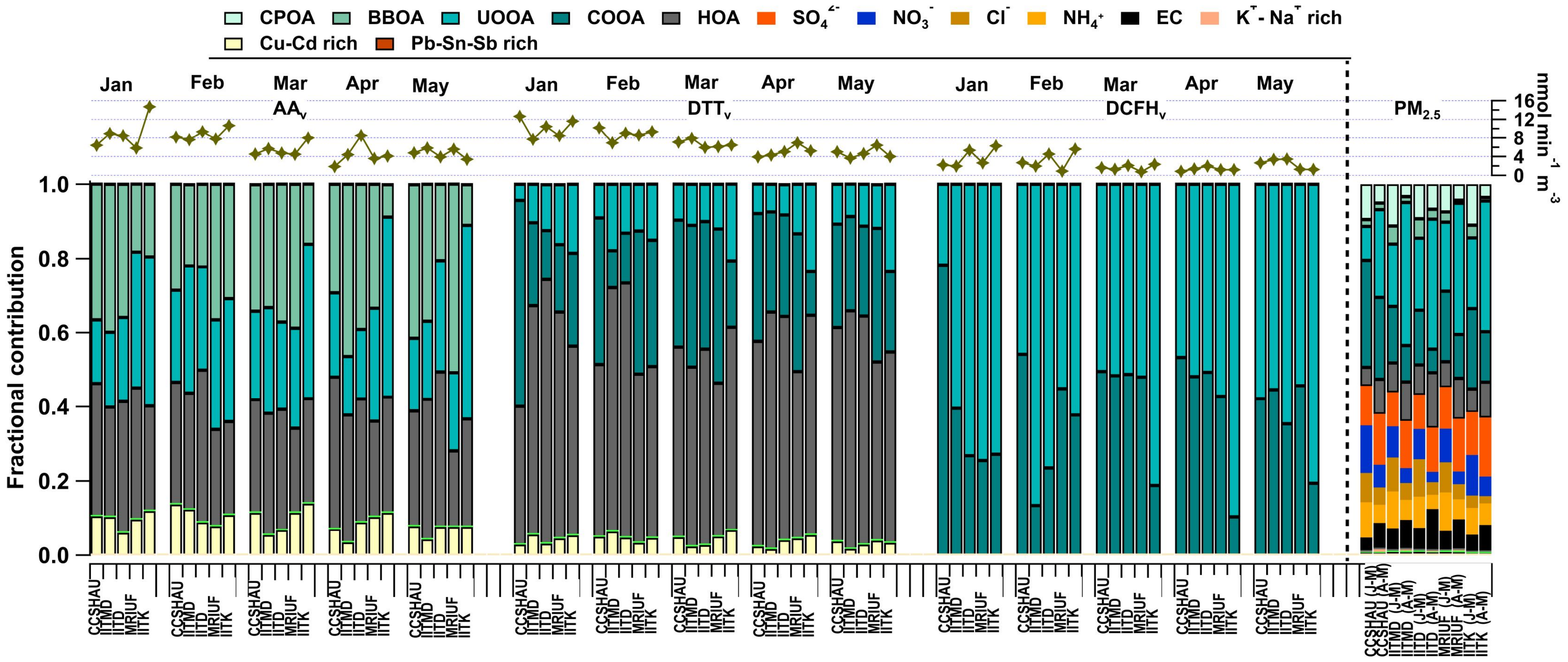

Supplementary Fig. 4: Comparison of source contribution to $PM_{2.5}$ and its OP_v. Site-specific monthly averaged concentration and fractional contribution of OA and elemental sources and total inorganics to total $PM_{2.5}$ and OP_v (AA, DTT and DCFH). The fractional contribution is estimated by considering the median OP_m of each source for each assay obtained from 1000 bootstrap runs while performing multi-linear regression, and OA sources as well as $PM_{2.5}$ spatial and temporal concentrations.

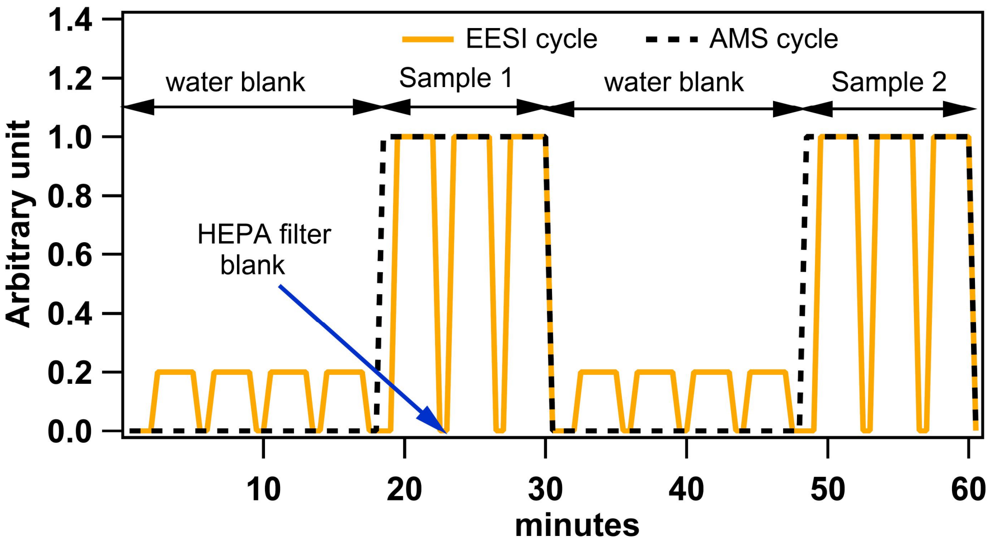

Supplementary Fig. 5: Typical sample measurement cycle of HR-AMS and EESI-ToF-MS. Each sample was measured for 12 minutes with 18 minutes of preceding water blank spending a total of 30 minutes on each sample by AMS and EESI. During EESI measurements, intermittent HEPA filter switching was performed to determine interferences due to sticky residuals from the previous sample.

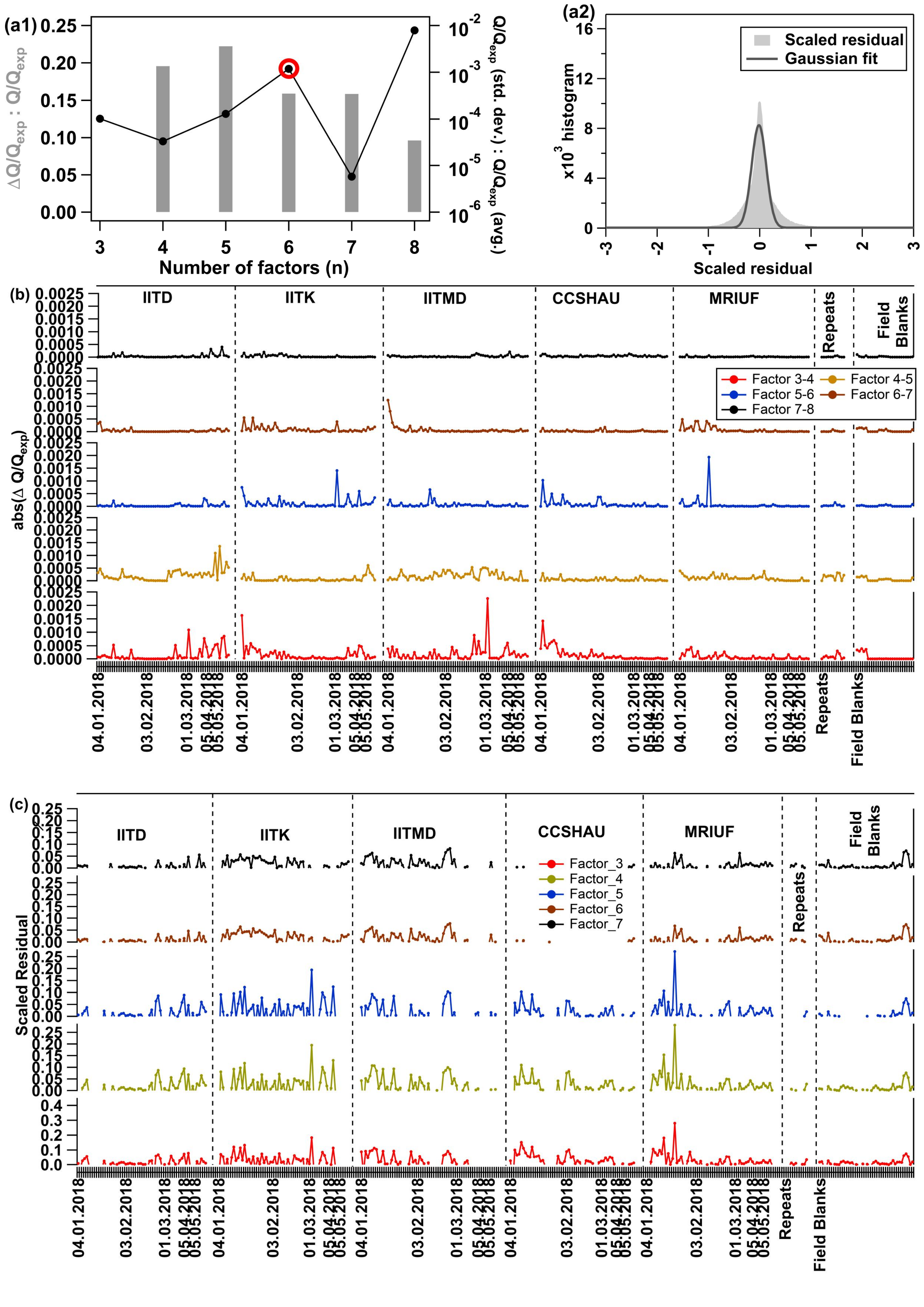

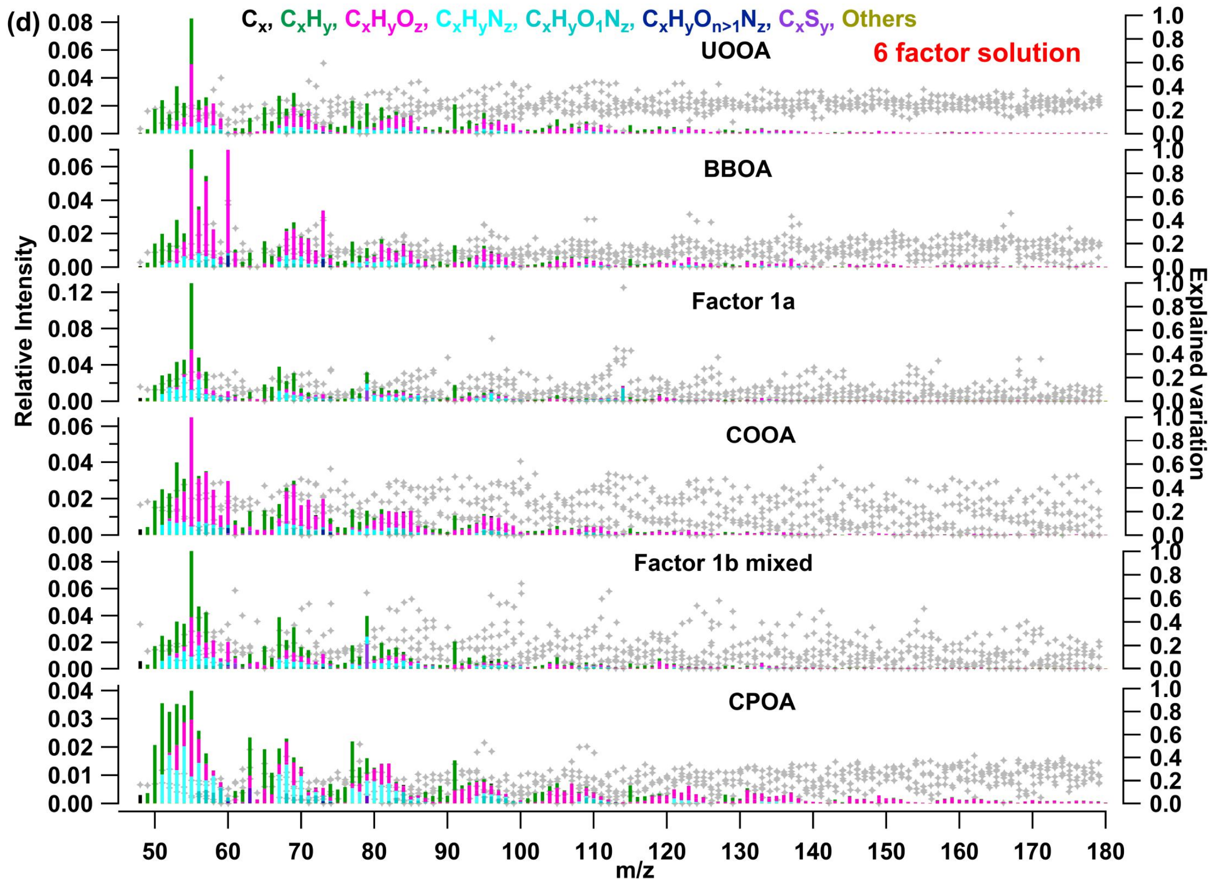
(d)
Cx, CxHy, CxHyOz, CxHyNz, CxHyO1Nz, CxHyOn>1Nz, CxSy, Others
6 factor solution
UOOA
BBOA
Factor 1a
COOA
Factor 1b mixed
CPOA
Relative Intensity
Explained variation
m/z

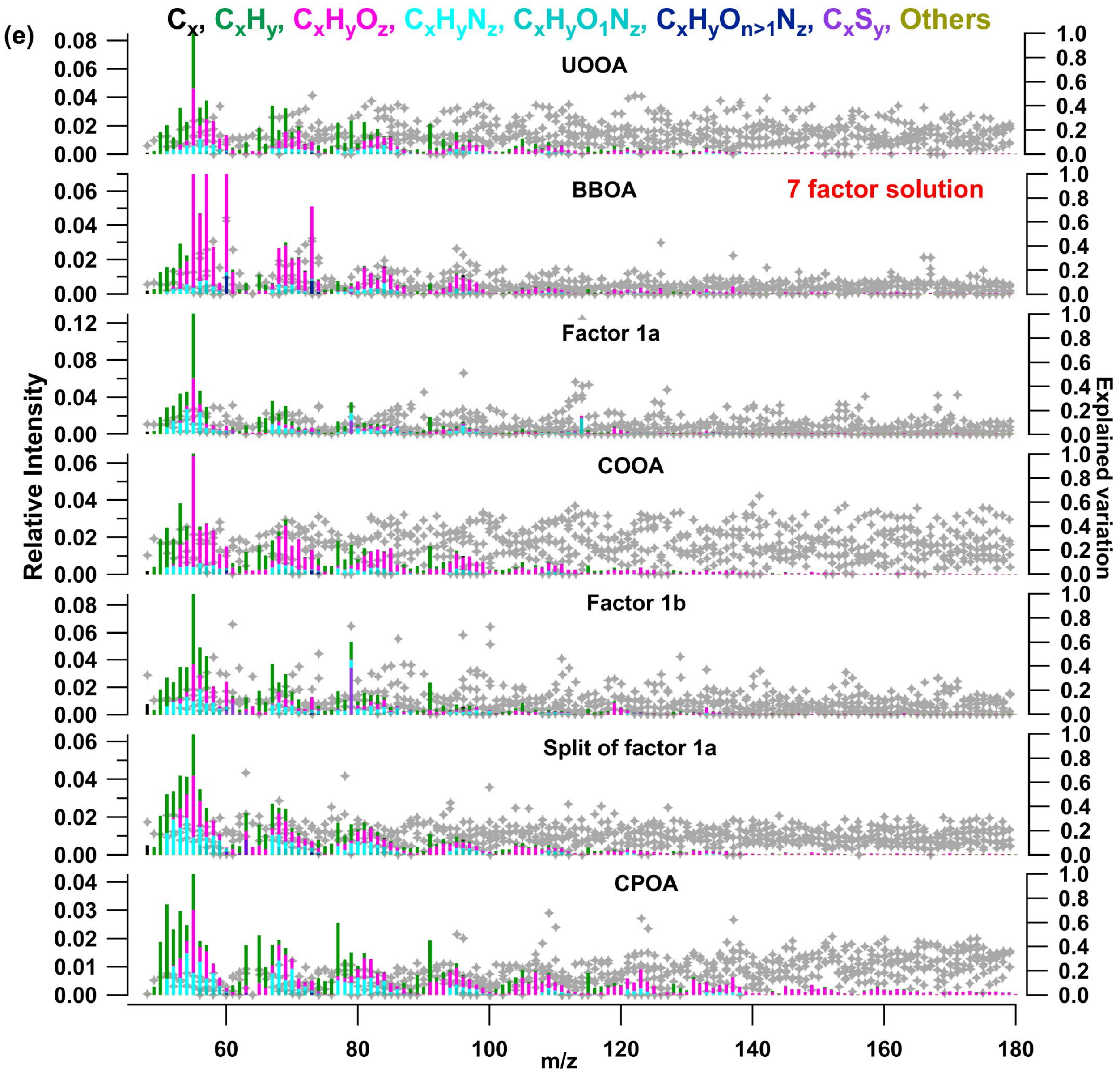
(e)
Cx, CxHy, CxHyOz, CxHyNz, CxHyO1Nz, CxHyOn>1Nz, CxSy, Others
7 factor solution
UOOA
BBOA
Factor 1a
COOA
Factor 1b
Split of factor 1a
CPOA
Relative Intensity
Explained variation
m/z

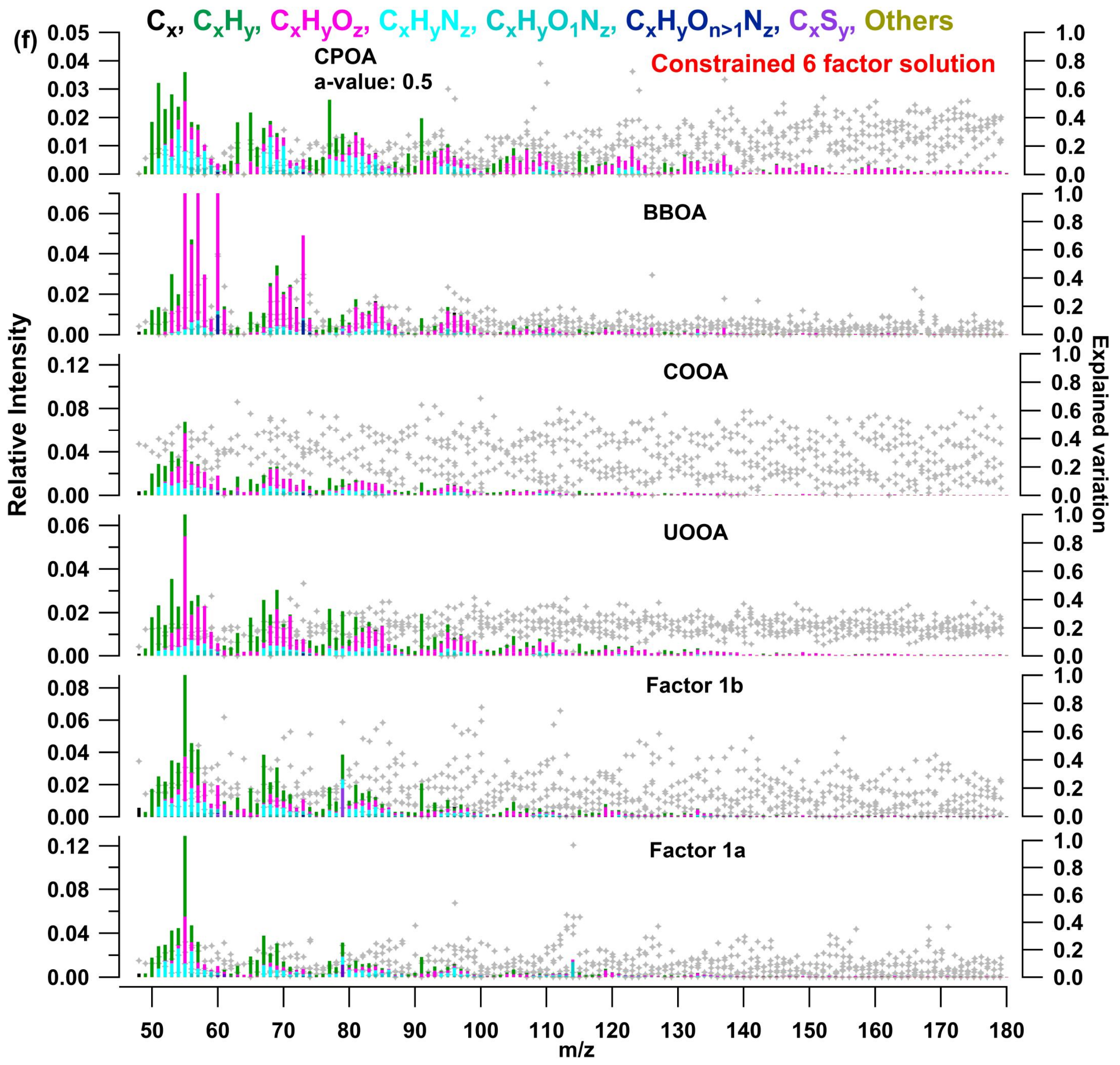
(f)
Cx, CxHy, CxHyOz, CxHyNz, CxHyO1Nz, CxHyOn>1Nz, CxSy, Others
CPOA
a-value: 0.5
Constrained 6 factor solution
BBOA
COOA
UOOA
Factor 1b
Factor 1a
Relative Intensity
Explained variation
m/z

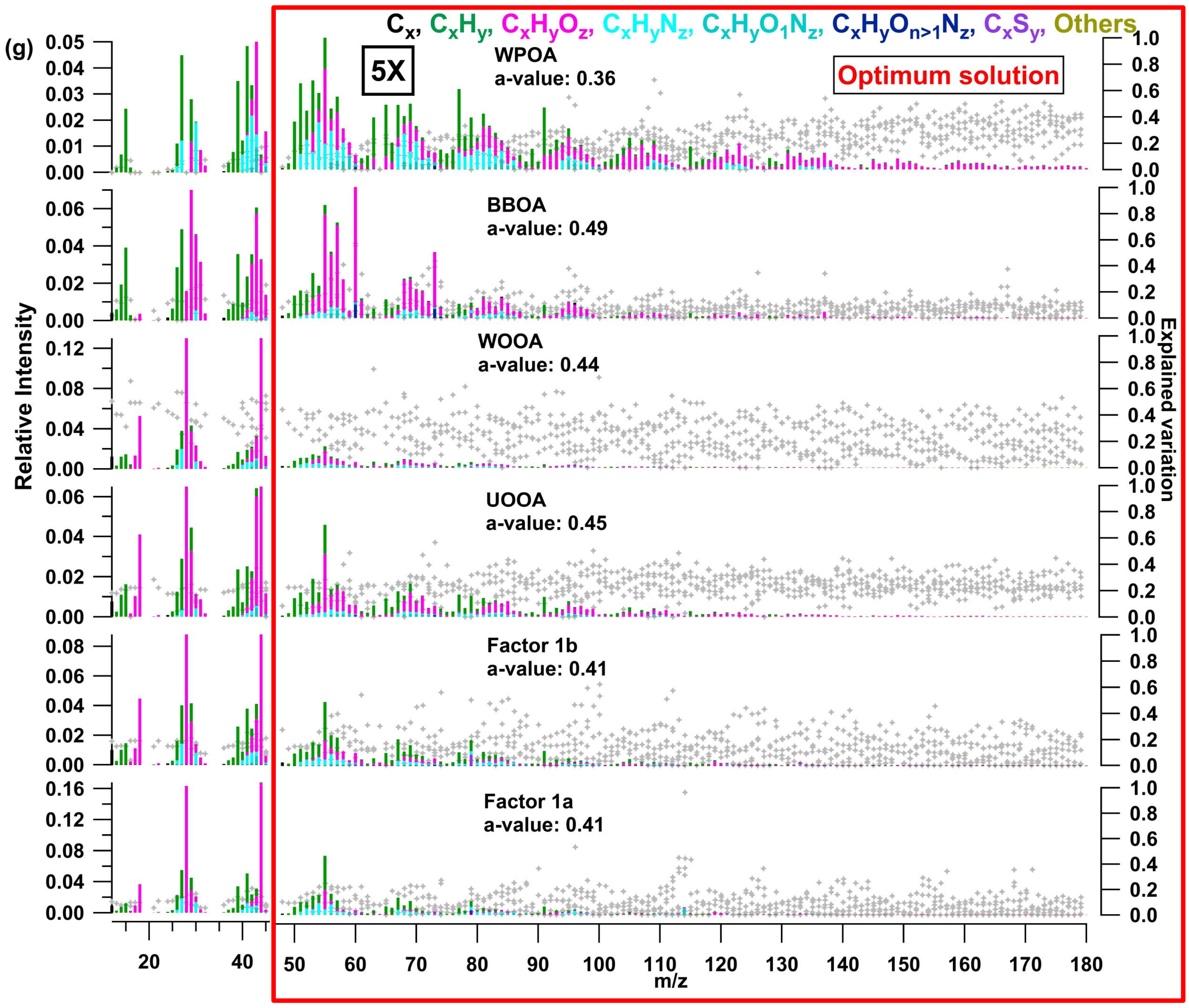
(g)
Cx, CxHy, CxHyOz, CxHyNz, CxHyO1Nz, CxHyOn>1Nz, CxSy, Others
5X
WPOA
a-value: 0.36
Optimum solution
BBOA
a-value: 0.49
WOOA
a-value: 0.44
UOOA
a-value: 0.45
Factor 1b
a-value: 0.41
Factor 1a
a-value: 0.41
Relative Intensity
Explained variation
m/z

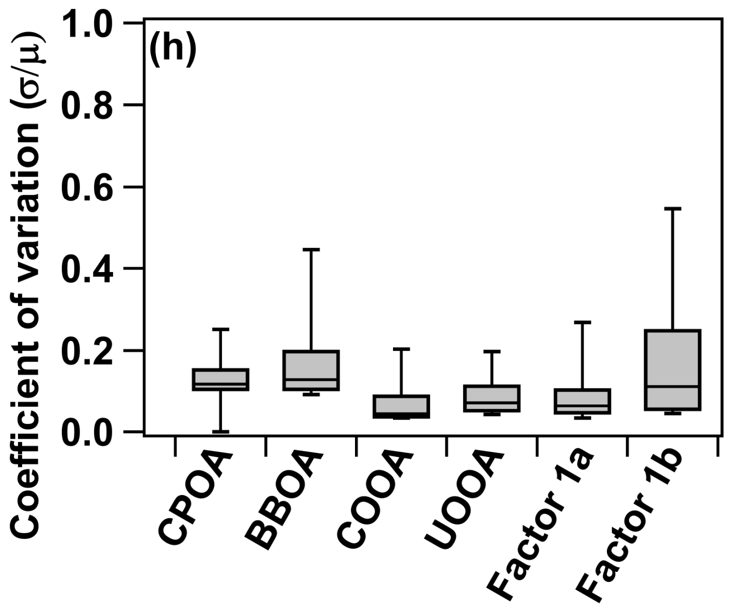
(h)
Coefficient of variation (σ/μ)
1.0
0.8
0.6
0.4
0.2
0.0
CPOA
BBOA
COOA
UOOA
Factor 1a
Factor 1b

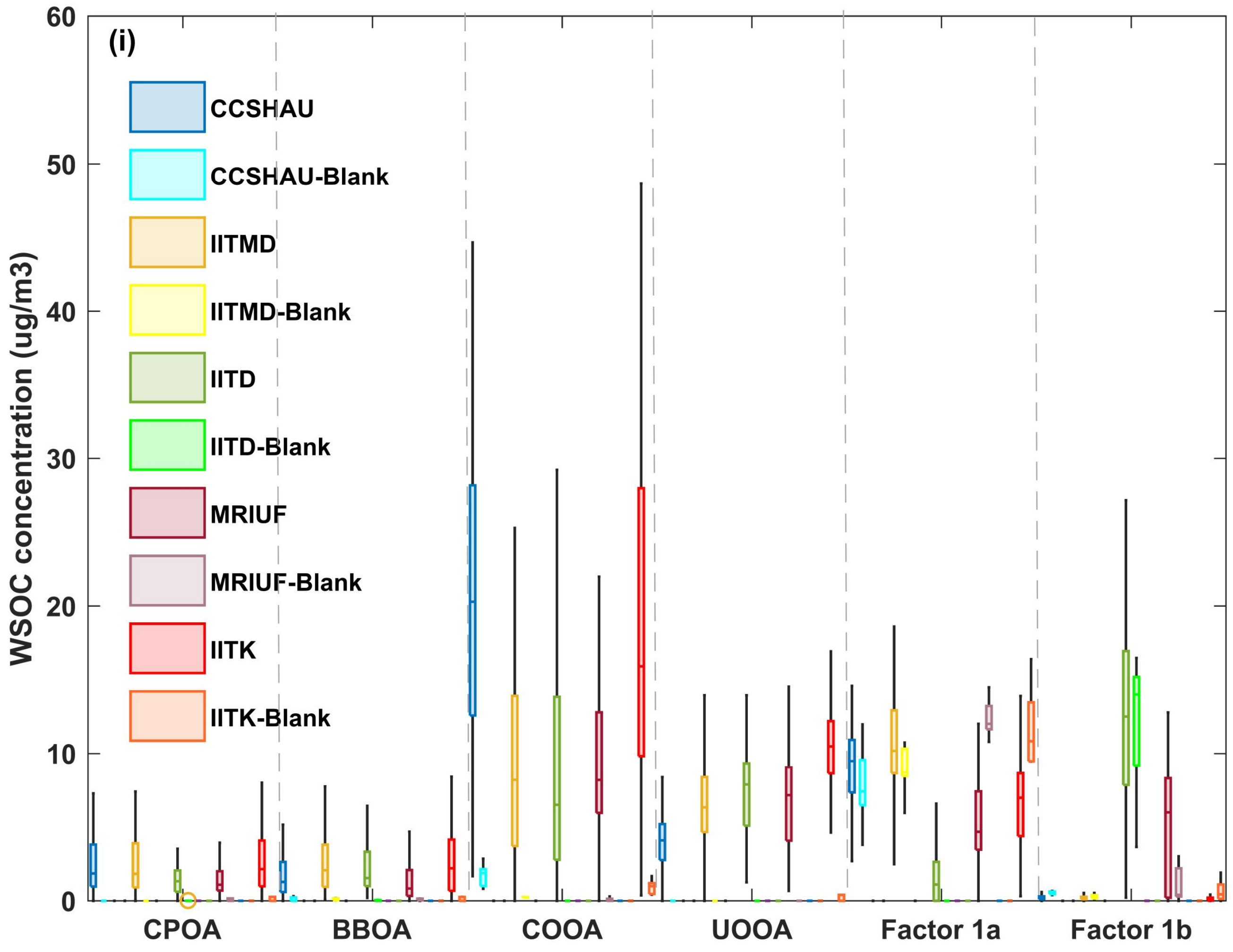
(i)
CCSHAU
CCSHAU-Blank
IITMD
IITMD-Blank
IITD
IITD-Blank
MRIUF
MRIUF-Blank
IITK
IITK-Blank
WSOC concentration (ug/m3)
60
50
40
30
20
10
0
CPOA
BBOA
COOA
UOOA
Factor 1a
Factor 1b

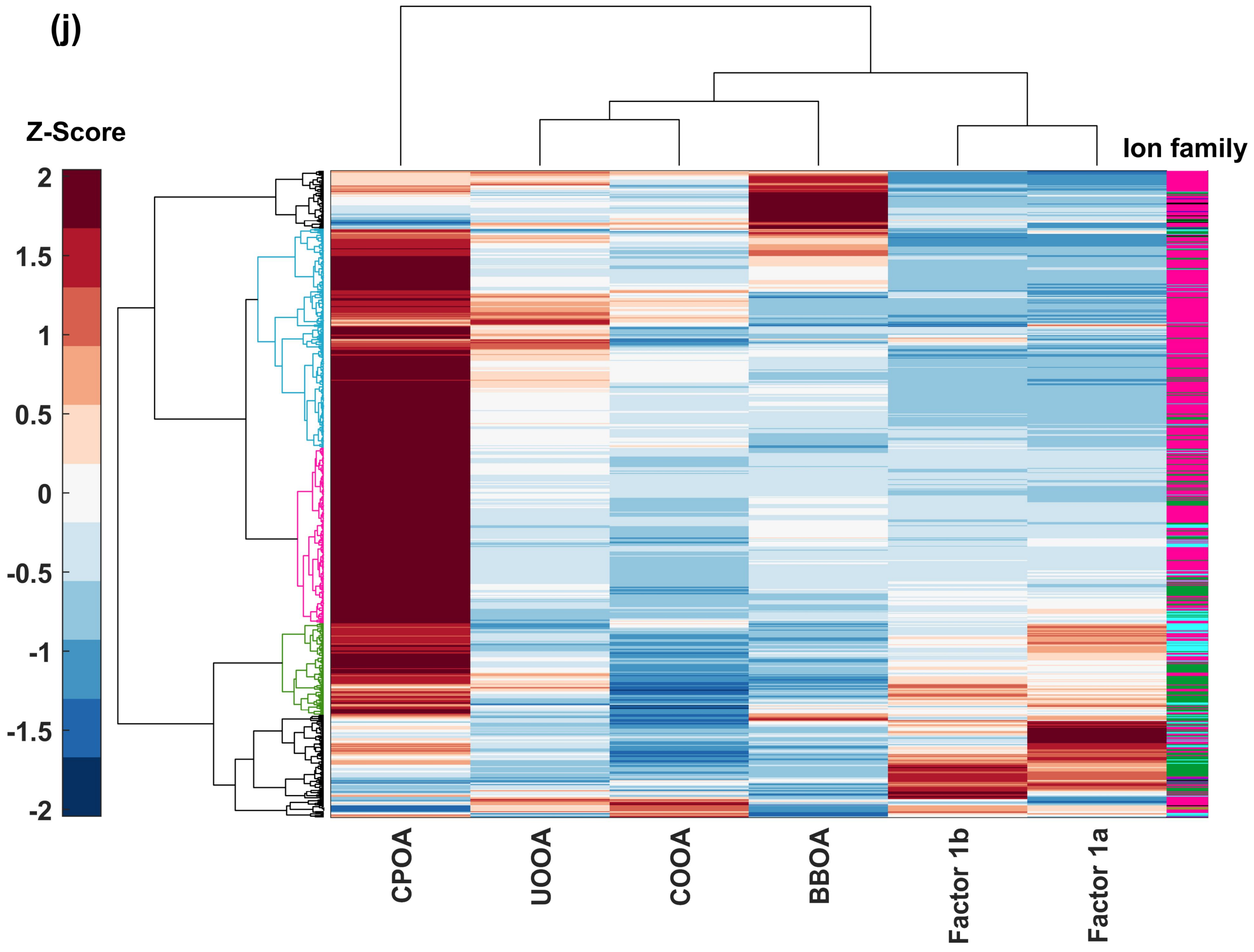
(j)
Z-Score
2
1.5
1
0.5
0
-0.5
-1
-1.5
-2
Ion family
CPOA
UOOA
COOA
BBOA
Factor 1b
Factor 1a

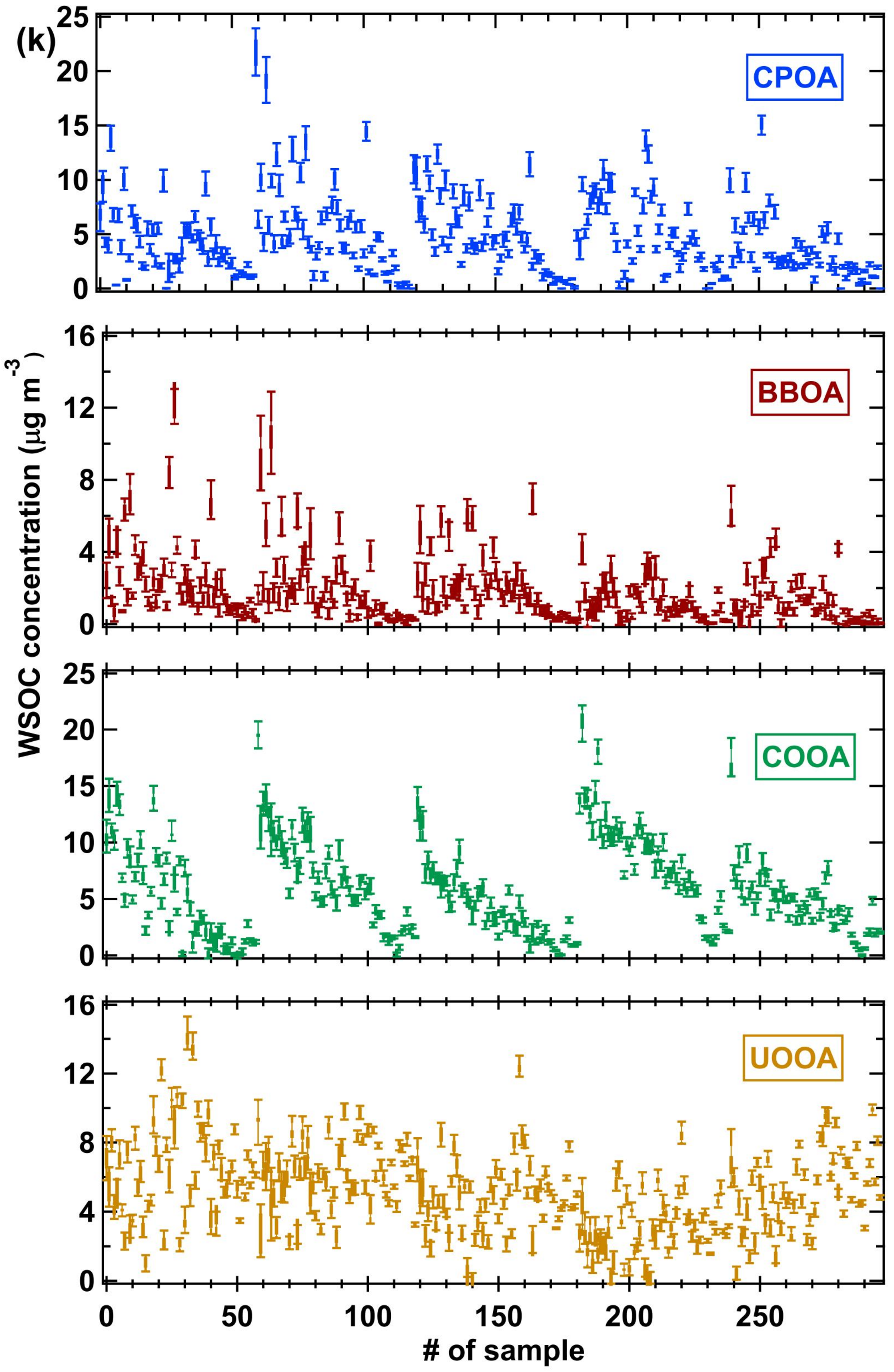

Supplementary Fig. 6: HR-AMS PMF diagnostics. a1) Diagnostic plot showing the relative change in Q/Q_{exp} and relative standard deviation for unconstrained solution vs. number of factors, respectively, and **a2)** Scaled residual distribution for the chosen 6-factor solution.s B) Absolute difference in $Q/Qexp$ between different number of factor solutions. **c)** Time series of scaled residuals for different number of factors (3-7). Factor profiles for unconstrained (m/z > 44) 6- **(d)** and 7-factor **(e)** solution. **f)** Factor profiles for 6-factor solution constrained using the CPOA profile (*a*-value = 0.5). **g)** Averaged (10 seed runs) optimum 6-factor solution (m/z 12-180) constrained with CPOA profile and passing upper and lower limits of the factor timeseries obtained from 100 BS runs of constrained PMF of HR ions with m/z > 44 **(e)**. **h)** Box and whisker plot of the Coefficient of variation (CoV) obtained for 100 BS runs of the optimum AMS-PMF solution obtained in **(g)**. **i)** Box whisker plot of water-soluble fraction of identified factors for all five sites. Unlike other factors, the OC concentrations of the contamination factors (Factor 1a and Factor 1b) have a similar range for field samples and field blanks. To identify unique ions related to single factors or a group of factors, 2D-hierarchical clustering was performed on AMS matrix of PMF (optimum solution shown in **g**) profiles and standardization (z-score) was done along the ions. A two-step clustering process (MATLAB 2016a); first along the columns (factors) and then the rows (ions) was done, where (i) the Euclidean distance was calculated for similarity/dissimilarity between pairs of ions/factors and (ii) then the Ward's method was used to form the linkages of ion/factor pairs. This resulted into **j)** dendograms with clusters of AMS ions on the y-axis (categorized into traditional AMS ion families with color scheme similar to **g)** and AMS-factors on the x-axis. It is interesting to note that CPOA is not only dominated by the CH and CHN ion groups (green cluster) but includes the majority of the fitted ions (CHOgt1 family in pink cluster and CHOgt1 and CHNO families in dark cyan cluster). The contamination factors (Factor 1a and 1b) are clustered together and the factors (UOOA and COOA) identified in the AMS-PMF on the basis of seasonal variation are clustered separately (see Materials and Methods). **k)** AMS measured-raw water-soluble mass concentration of each factor across all sites. Box whisker plot represent values obtained from 100 bootstrap runs.

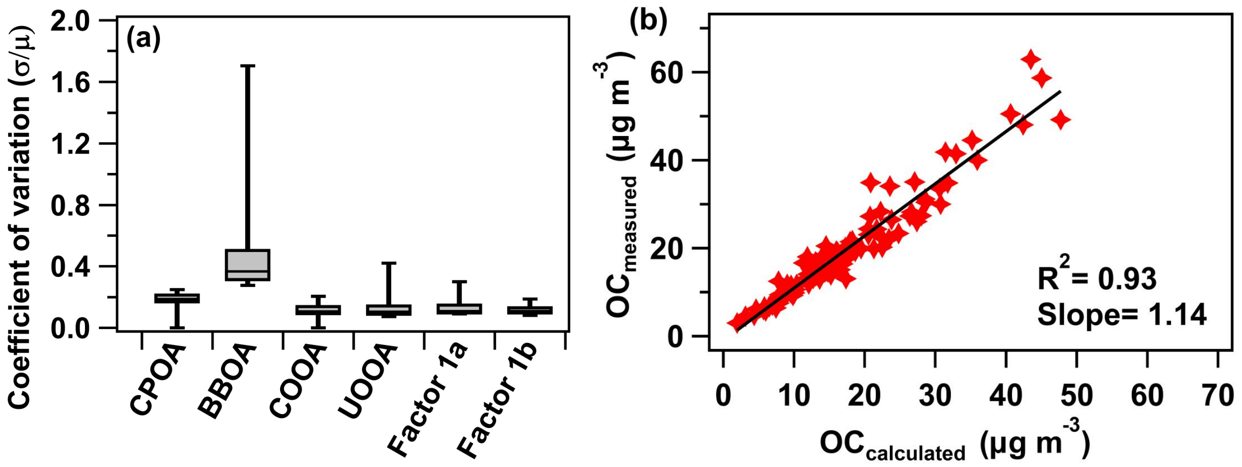

Supplementary Fig. 7: Species-specific factor recoveries. (a) Box and whisker plot (10th, 25th, 50th, 75th and 90th percentile) of the Coefficient of variation (CoV) obtained for the optimum PMF solution for estimating species-specific factor recoveries. 100 BS runs were performed on water-soluble AMS ions and water-insoluble OC (WIOC) while constraining the solution with upper and lower concentration limits for each factor. The higher value for BBOA is observed in the runs where the sample concentration decreases by a factor of 100. **(b)** Mass closure of reconstructed recovery corrected AMS-PMF-derived OC mass and Sunset-measured OC mass.

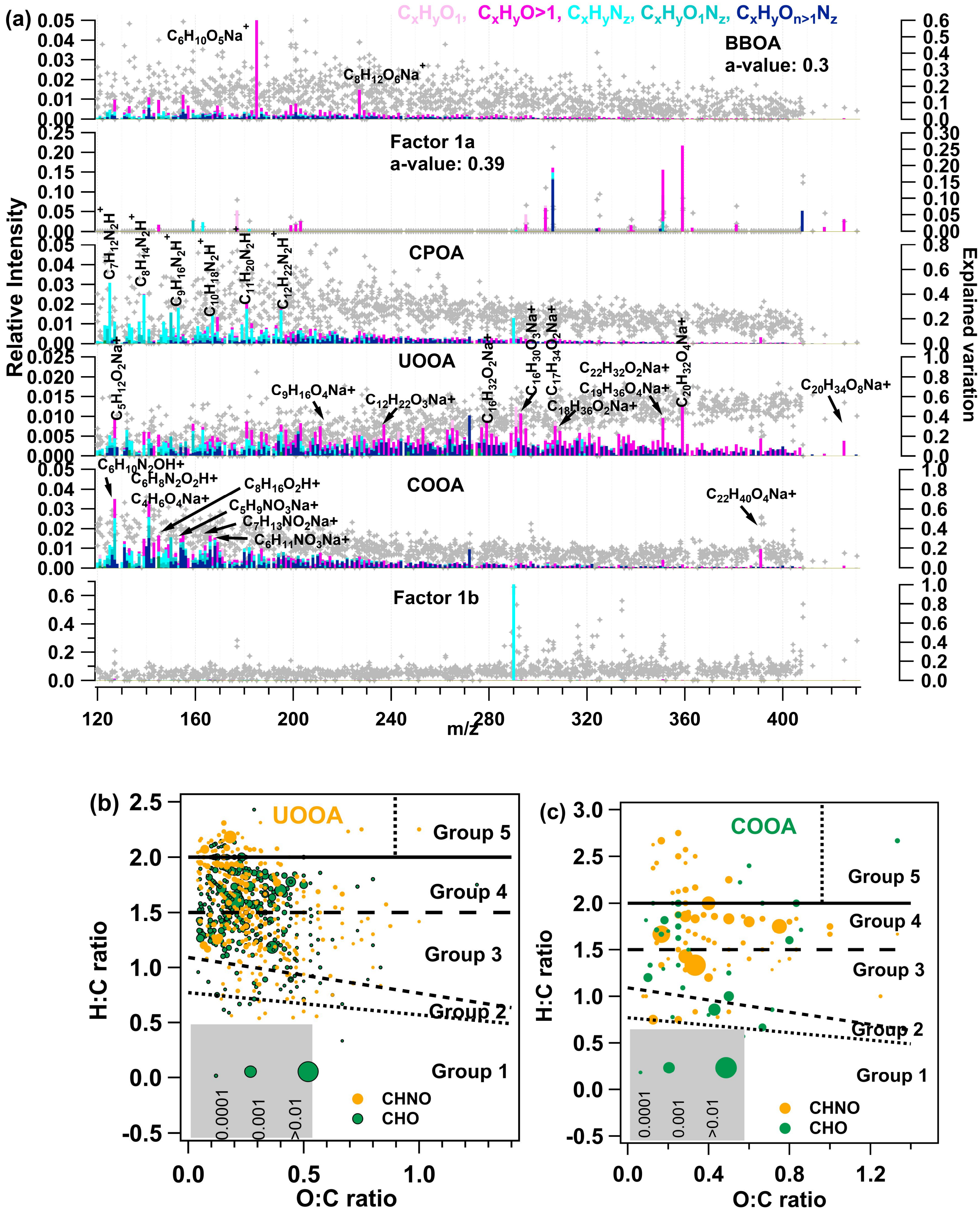

Supplementary Fig. 8: Near-molecular level chemical fingerprints of OA sources. (a) Averaged (10 seed runs) optimum 6-factor solution (*m/z* 120-440) constrained with BBOA profile (from 9-factor solution; *a* = 0.3) and AMS-derived time series of contamination factor-1b (*a* =0.39). The signal intensity (SI) of $C_6H_{10}O_5Na^+$ in all source profiles was reduced by a factor 10 to clearly show the other contributing marker ions. Further, atomic H:C vs O:C ratios of individual sources: **(b)** UOOA, and **(c)** COOA. The lines represent different groups defined on the basis of modified

aromaticity index (AI_{mod}) and H:C ratio where Group 1 representing combustion-derived condensed polycyclic aromatics ($AI_{mod} > 0.66$) lie below the dotted line, Group 2 representing vascular plant-derived polyphenols ($0.66 \geq AI_{mod} > 0.50$) lie between the dotted and short dashed line, Group 3 with highly unsaturated and phenolic compounds ($AI_{mod} \leq 0.50$ and H:C < 1.5) between the short dashed and medium dashed line, Group 4 of aliphatic compounds (2.0 ≥ H:C ≥ 1.5) is between the medium dashed and solid line, and Group 5 representing saturated fatty and carbohydrates (H:C > 2.0) lie above the solid line. Here, all ions having a relative contribution (f_c) of the factor greater than 0.35 were selected for COOA whereas for UOOA, those having f_c of UOOA greater than 0.4 and f_c of other remaining factors lower than 0.3, were selected. The marker size represents the selected ions' fractional contribution to the factor profile.

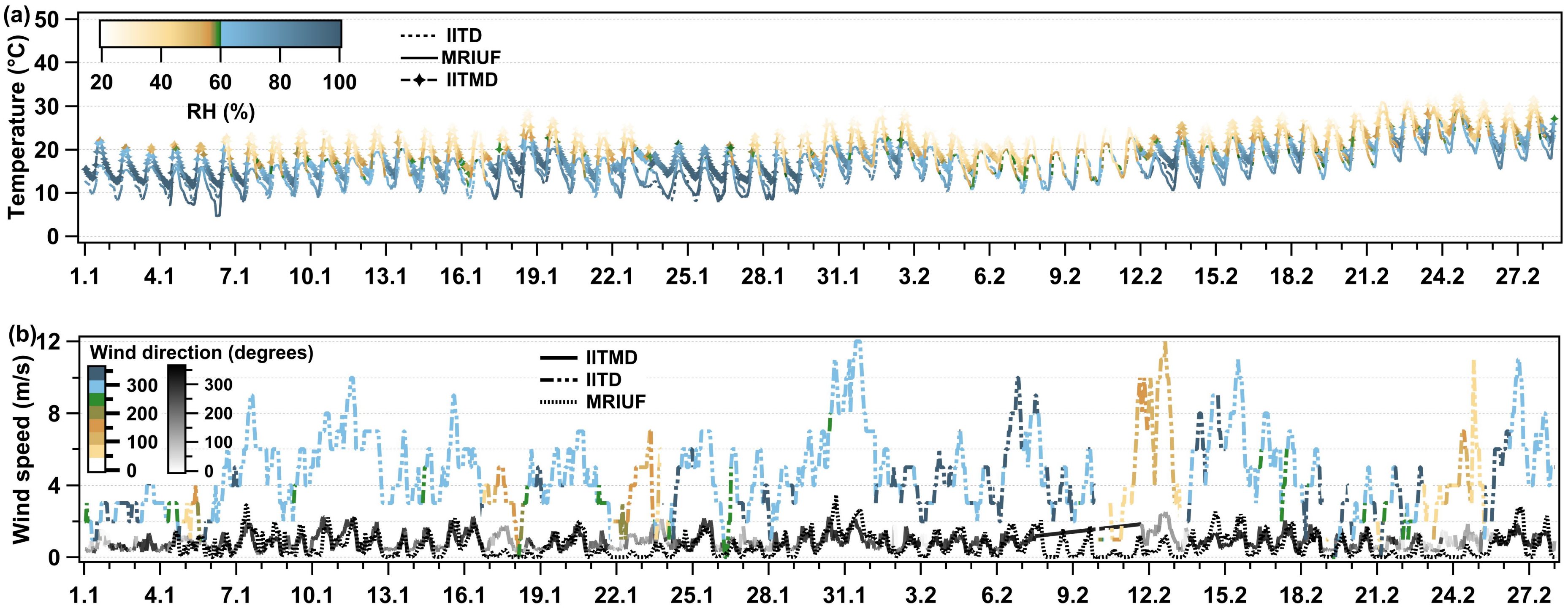

Supplementary Fig. 9: Meteorological parameters measured at three sites in Delhi. Temporal variation of **(a)** temperature (°C) and relative humidity (%), **(b)** wind speed (m s^{-1}) and wind direction (degrees) measured at IITD, IITMD (from the DPCC station at Mandir Marg, roughly 2.7 km air distance) and MRIUF from January to February, 2018 using automated weather station installed at the building rooftop.

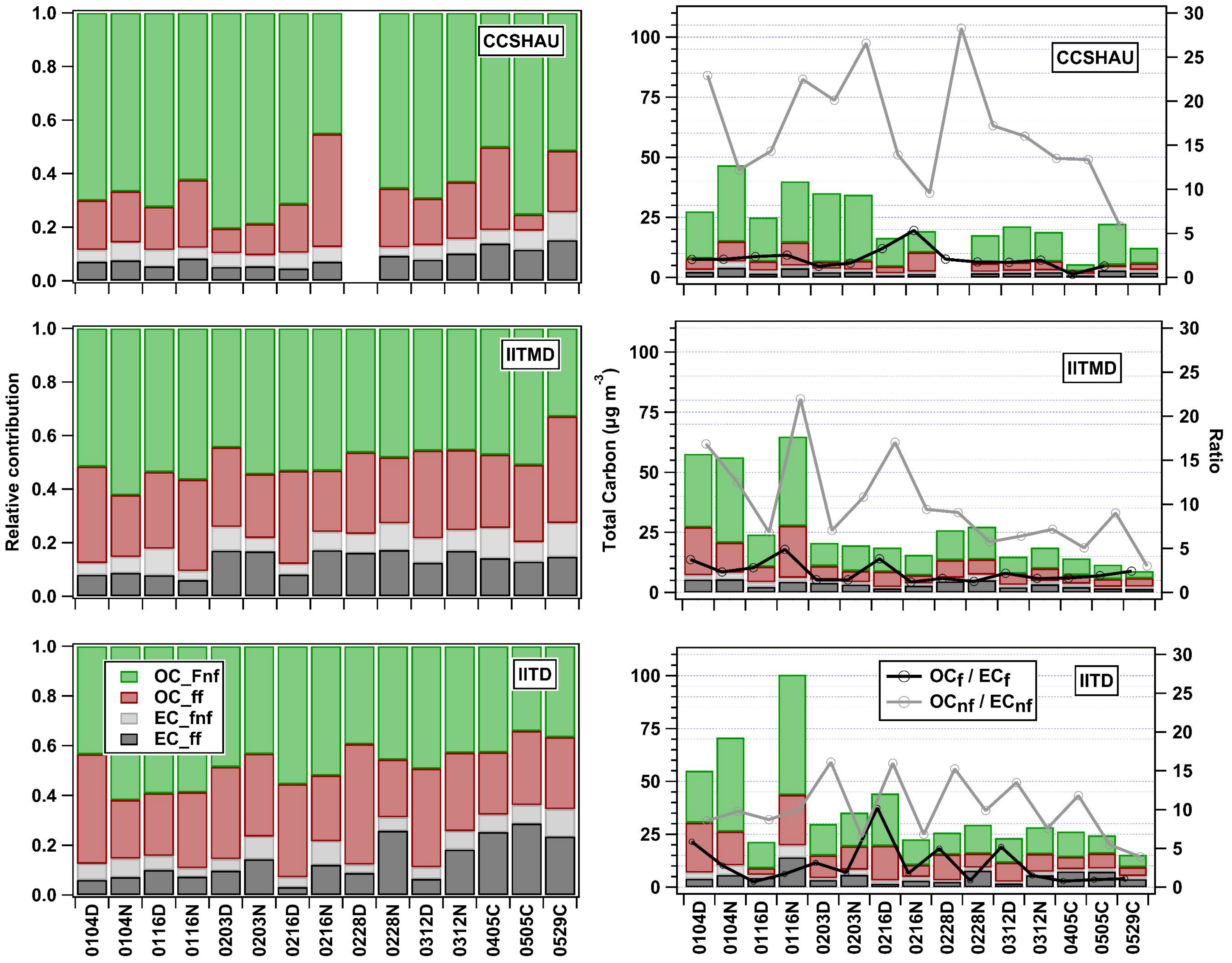

Supplementary Fig. 10: Fossil and non-fossil fraction of OC and EC. Relative contributions [left panels] and absolute concentrations [right panels] of the fossil and non-fossil fractions of OC and EC at three sites (CCSHAU, IITMD and IITD).

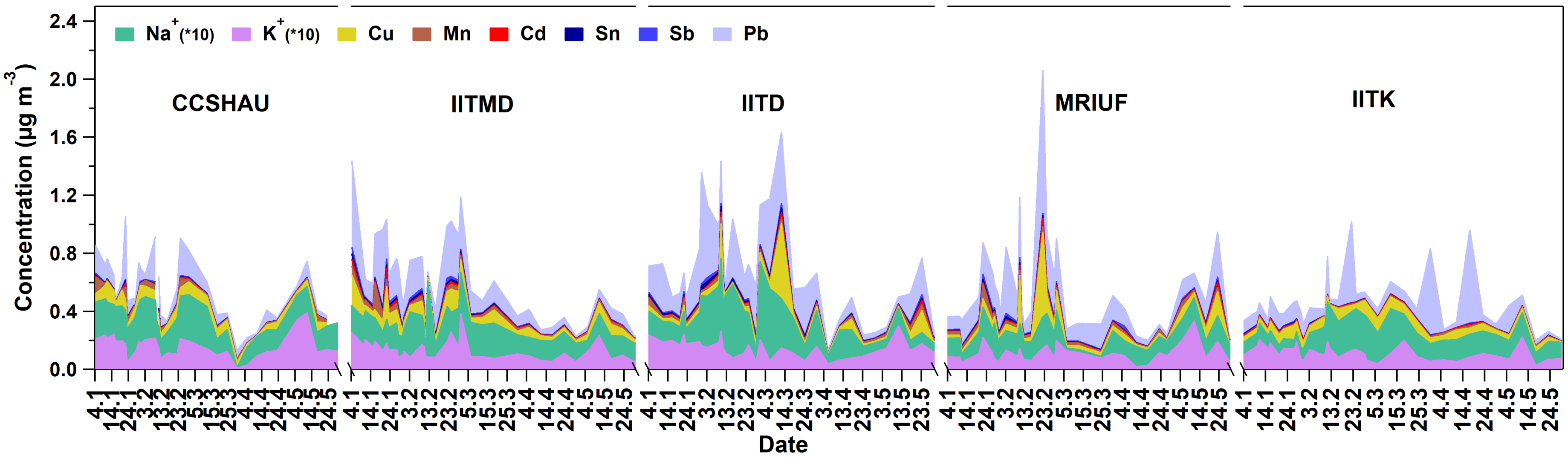

Supplementary Fig. 11: Total elemental concentration and its components. (A) Spatially resolved (all 5 sites) stacked time series of trace elements (except contaminated ones) and water-soluble marker ions. The trace elements include Cu, Mn, Cd, Sn, Sb, and Pb measured by ICP-MS. Na^+ and K^+ (here, divided by a factor of 10 for demonstration purpose) were the two water-soluble marker ions measured by ion chromatography.

344

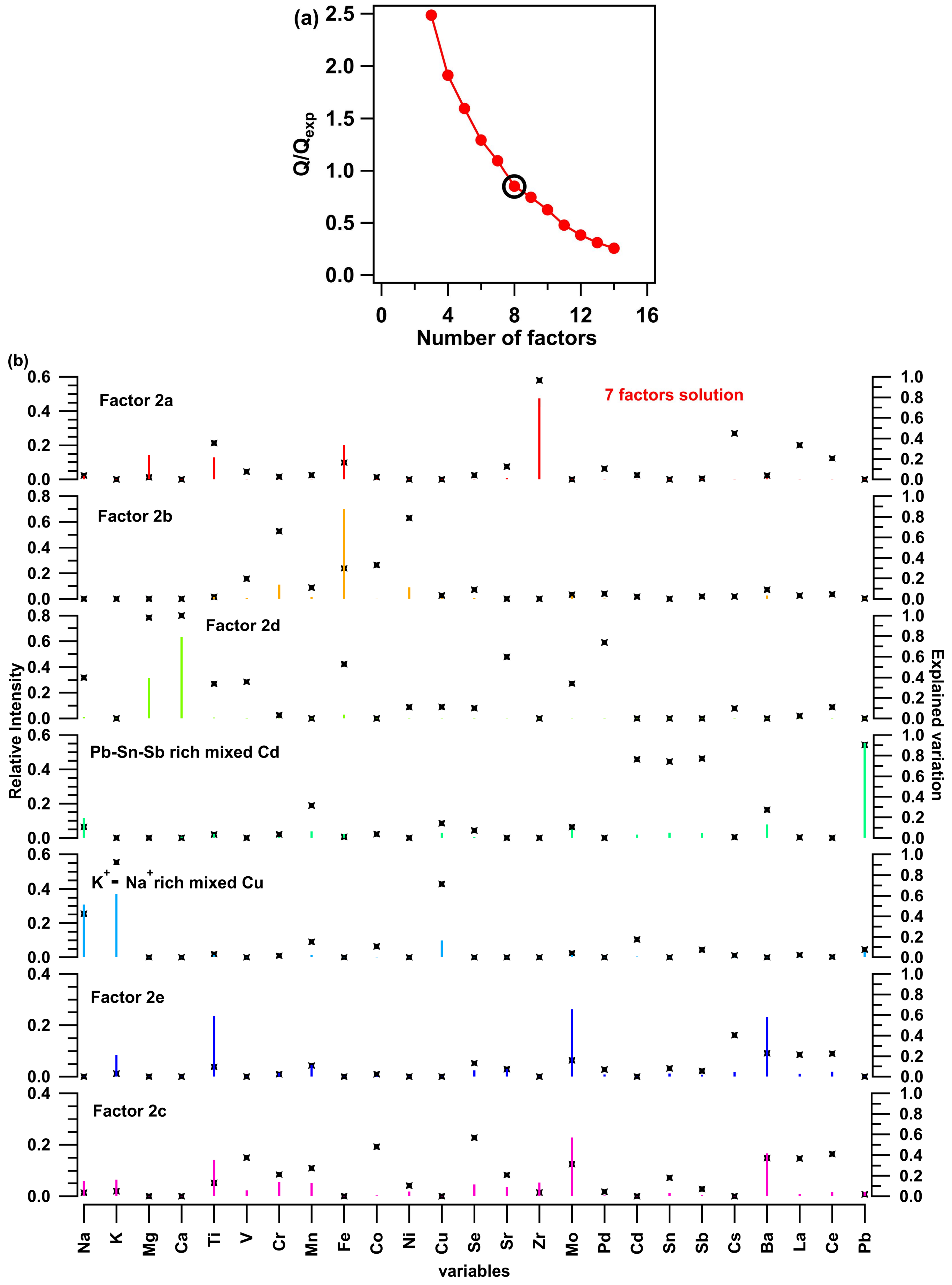

(a)
Q/Q_exp
Number of factors
(b)
7 factors solution
Factor 2a
Factor 2b
Factor 2d
Pb-Sn-Sb rich mixed Cd
K+- Na+ rich mixed Cu
Factor 2e
Factor 2c
Relative Intensity
Explained variation
Na
K
Mg
Ca
Ti
V
Cr
Mn
Fe
Co
Ni
Cu
Se
Sr
Zr
Mo
Pd
Cd
Sn
Sb
Cs
Ba
La
Ce
Pb
variables

345

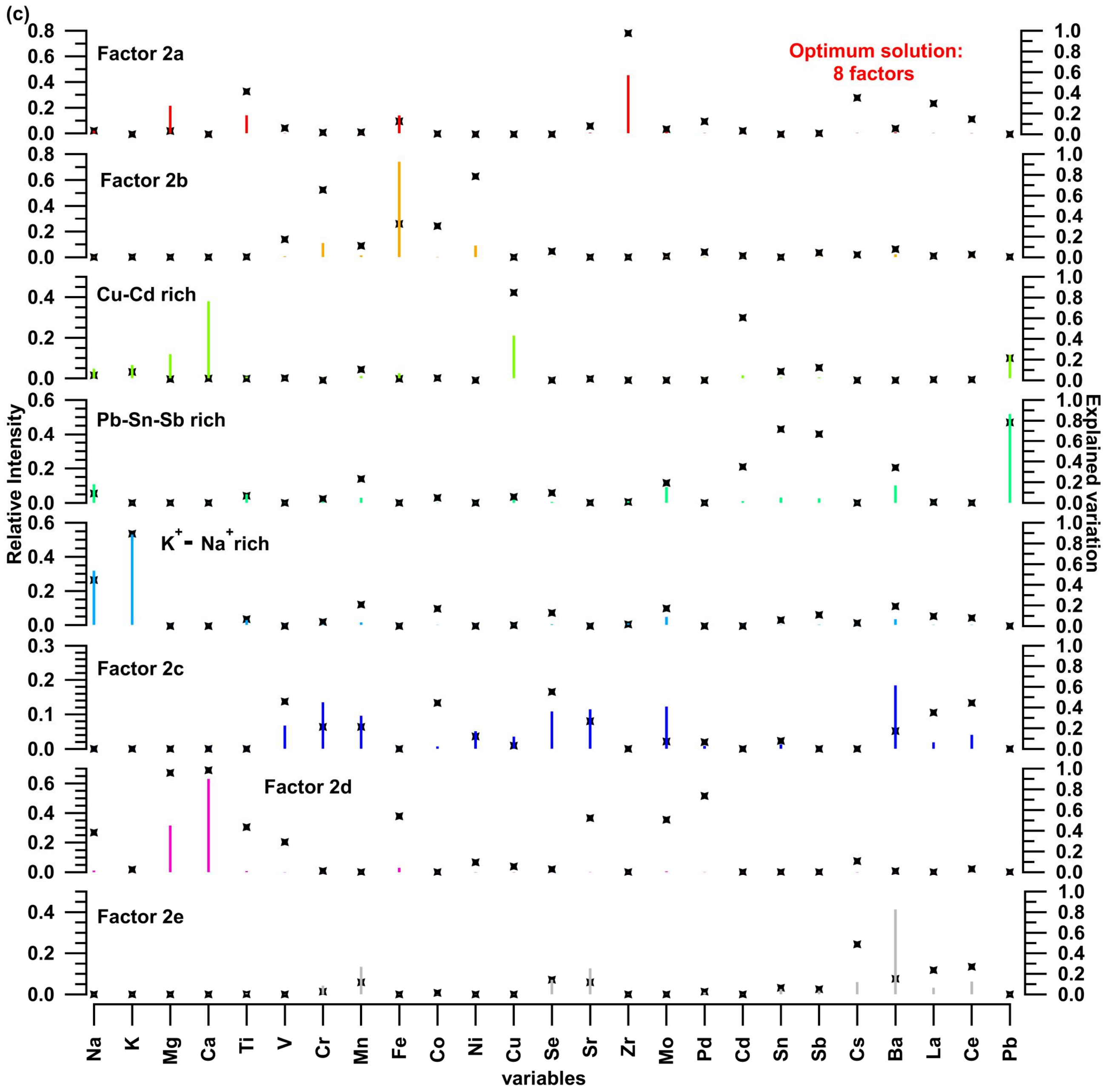

(c)
Optimum solution:
8 factors
Factor 2a
Factor 2b
Cu-Cd rich
Pb-Sn-Sb rich
K^+- Na^+rich
Factor 2c
Factor 2d
Factor 2e
Relative Intensity
Explained variation
Na K Mg Ca Ti V Cr Mn Fe Co Ni Cu Se Sr Zr Mo Pd Cd Sn Sb Cs Ba La Ce Pb
variables

346

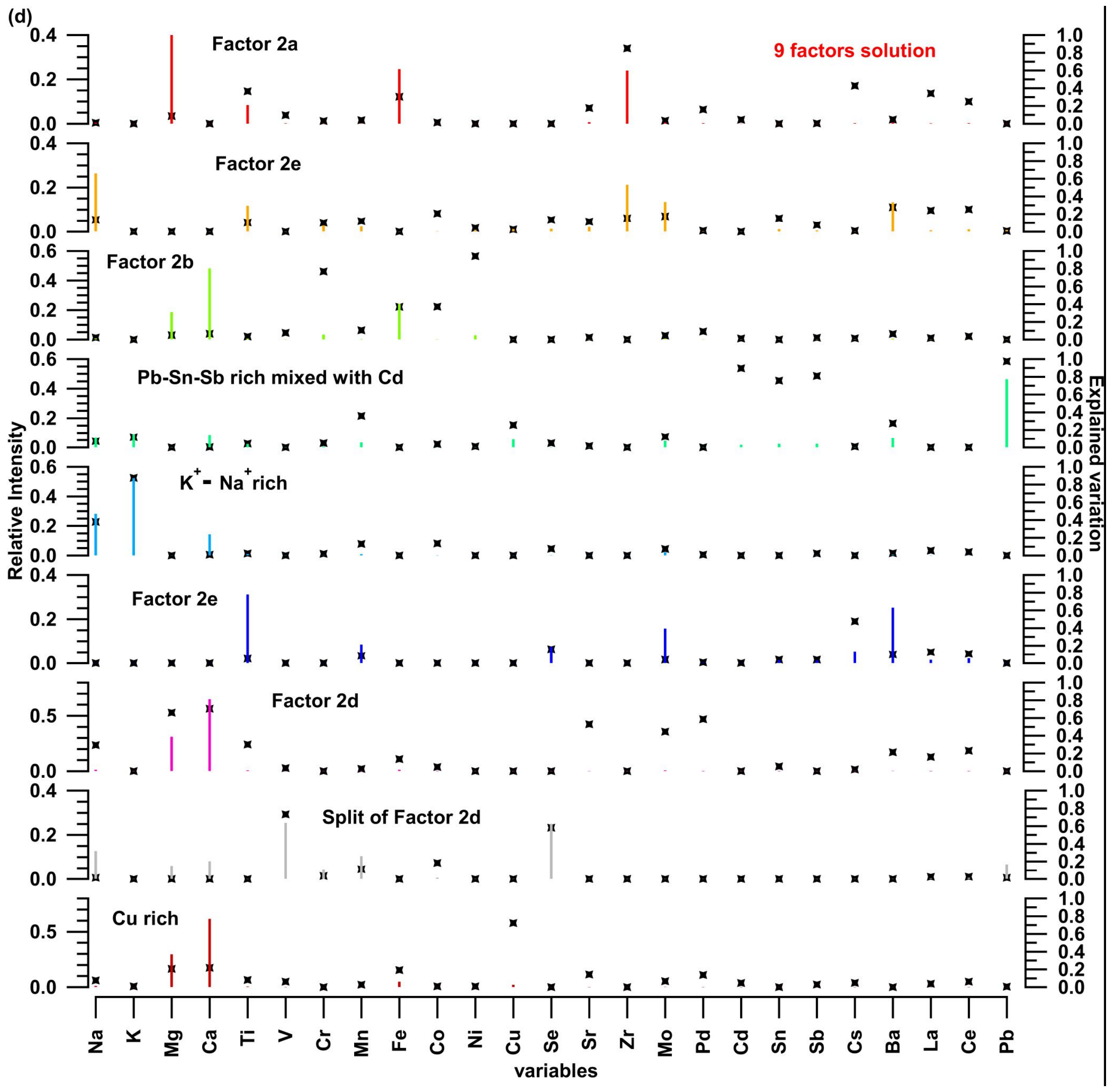

(d)
9 factors solution
Factor 2a
Factor 2e
Factor 2b
Pb-Sn-Sb rich mixed with Cd
K^+- Na^+rich
Factor 2e
Factor 2d
Split of Factor 2d
Cu rich
Relative Intensity
Explained variation
Na K Mg Ca Ti V Cr Mn Fe Co Ni Cu Se Sr Zr Mo Pd Cd Sn Sb Cs Ba La Ce Pb
variables

347

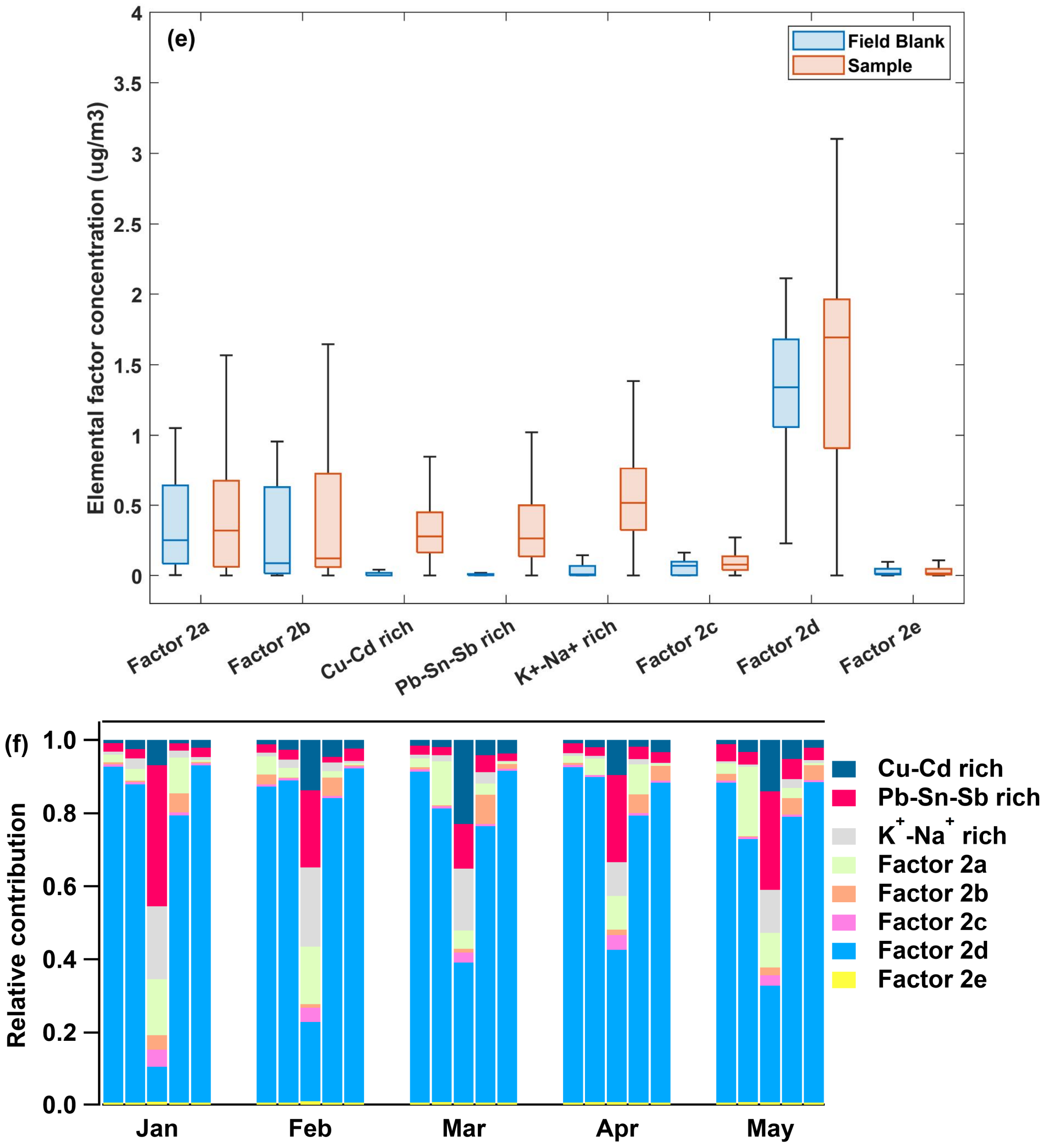

Supplementary Fig. 12: Sources of trace elements. Unconstrained PMF of 27 elements, including field blanks and aerosol samples. **(a)** Diagnostic plot showing the relative change in $Q/Q_{exp.}$ **(b)** Factor profiles for 7-factors solution with Pb-Sn-Sb rich mixed with Cd and K^+-Na^+ mixed with Cu. **(c)** Factor profiles for optimum 8-factor solution. **(d)** Factor profiles for 9-factors solution with Pb-Sn-Sb rich mixed with Cd and splitting of Factor 2d. **(e)** Box whisker plot of elemental sources concentrations in field and blank samples of all five sites. **(f)** Relative contribution of elemental sources.

Supplementary Tables

Supplementary Table 1. Comparison of wintertime total OP_v (AA, DTT), $PM_{2.5}$ mass and $PM_{2.5}$ oxidative strength across different regions of the world.

Sampling site	Period	Site classification	Number of samples	OP_m (AA, DTT; nmol min^{-1} ug^{-1})	OP_v (AA, DTT; nmol min^{-1} m^{-3})	$PM_{2.5}$ (µg m^{-3})
IITD, Delhi, India	2018	Traffic	55	0.08, 0.09	7.8, 8.5	115
	2019	Traffic	105	0.14, 0.10	14.4, 9.5	94
IITK, Kanpur (downwind of Delhi), India	2018	Sub-urban background	57	0.12, 0.10	10.8, 9.3	99
CCSHAU (upwind of Delhi), Haryana, India	2018	Rural/background	57	0.07, 0.11	6.4, 10.2	105
IITMD, Delhi, India	2018	Urban/background	61	0.09, 0.09	7.4, 7.1	85
MRIU Faridabad, Haryana, India	2018	Sub-urban/industrial	56	0.07, 0.10	6.1, 8.4	104
Palau Reial, Barcelona, Spain	2018-2019	Urban	114	0.06, 0.07	1.0, 1.2	18
Bure, France	2014-2015	Rural	102	0.03, 0.05	0.2, 0.5	9
Chongqing, China	2020	Urban	113	0.07, 0.07	3.7, 3.7	54
Xi'an, China	2020	Urban	130	0.07, 0.09	5.5, 8.0	87
Bern, Switzerland	2018-2020	Traffic	176	0.13, 0.09	1.4, 1.1	12

Magadino, Switzerland	2018-2019	Alpine valley	90	0.10, 0.06	1.2, 0.7	10
Zurich-Kaserne, Switzerland	2018-2019	Urban	90	0.08, 0.08	0.9, 0.8	11
Payerne, Switzerland	2018-2020	Rural	91	0.04, 0.06	0.4, 0.6	9

Supplementary Table 2. OA sources vs. targeted organic compounds correlation matrix. Fossil (*f*) and non-fossil *(nf)* fraction of OA sources are correlated to organic markers measured by GC-MS and LC-MS.

	$CPOA_f$	$BBOA_f$	$COOA_f$	$UOOA_f$	HOA_{est}	$CPOA_{nf}$	$BBOA_{nf}$	$COOA_{nf}$	$UOOA_{nf}$
Maleic acid	0.31	0.14	0.37	-0.27	-0.04	0.31	0.14	0.37	-0.27
Succinic acid	0.44	0.42	0.75	-0.13	0.19	0.44	0.42	0.75	-0.13
Citraconic acid	0.53	0.46	0.80	-0.09	0.28	0.53	0.46	0.80	-0.09
Glutaric acid	0.50	0.53	0.62	0.01	0.39	0.50	0.53	0.62	0.01
Oxoheptanedioic acid	0.11	0.02	0.22	0.32	0.25	0.11	0.02	0.22	0.32
MethylSuccinic acid	0.55	0.54	0.75	-0.11	0.29	0.55	0.54	0.75	-0.11
Adipic acid	0.59	0.54	0.69	0.03	0.35	0.59	0.54	0.69	0.03
Methylglutaric acid	0.51	0.45	0.72	0.03	0.30	0.51	0.45	0.72	0.03
Phtalic acid	0.47	0.41	0.66	0.15	0.26	0.47	0.41	0.66	0.15
Suberic acid	0.52	0.48	0.63	0.00	0.16	0.52	0.48	0.63	0.00
Azelaic acid	0.64	0.63	0.52	0.03	0.33	0.64	0.63	0.52	0.03
Sébacic acid	0.62	0.49	0.67	-0.08	0.15	0.62	0.49	0.67	-0.08
Fluoranthene	0.76	0.75	0.54	0.03	0.45	0.76	0.75	0.54	0.03
Acephenanthrylene	0.73	0.74	0.55	-0.08	0.38	0.73	0.74	0.55	-0.08
Pyrene	0.75	0.74	0.51	0.04	0.49	0.75	0.74	0.51	0.04
Benzo[c]phenanthrene	0.57	0.40	0.45	-0.22	-0.04	0.57	0.40	0.45	-0.22
Benzo[ghi]fluoranthene	0.70	0.84	0.48	-0.30	0.42	0.70	0.84	0.48	-0.30
Benz[a]anthracene	0.62	0.86	0.32	-0.30	0.35	0.62	0.86	0.32	-0.30
Chrysene	0.76	0.79	0.51	-0.10	0.39	0.76	0.79	0.51	-0.10
sum_Benzo[b,k]fluoranthene	0.76	0.84	0.42	-0.05	0.47	0.76	0.84	0.42	-0.05
2,2'-Binaphthalene	0.56	0.74	0.08	-0.31	0.31	0.56	0.74	0.08	-0.31
Benz[e]pyrene	0.74	0.76	0.41	-0.02	0.47	0.74	0.76	0.41	-0.02
Benz[a]pyrene	0.63	0.84	0.28	-0.05	0.48	0.63	0.84	0.28	-0.05
Perylene	0.63	0.81	0.29	-0.06	0.45	0.63	0.81	0.29	-0.06
Anthanthrene	0.49	0.76	0.13	-0.03	0.43	0.49	0.76	0.13	-0.03
Dibenz[ah]anthracene	0.67	0.78	0.34	-0.01	0.47	0.67	0.78	0.34	-0.01
Indeno[1,2,3-cd]pyrene	0.71	0.79	0.35	-0.01	0.50	0.71	0.79	0.35	-0.01
Picene	0.59	0.74	0.30	-0.08	0.38	0.59	0.74	0.30	-0.08
Benzo[ghi]perylene	0.71	0.80	0.34	0.03	0.56	0.71	0.80	0.34	0.03
Coronene	0.61	0.74	0.25	0.06	0.55	0.61	0.74	0.25	0.06
Naphtho[1,2-kb]fluoranthene	0.66	0.74	0.34	-0.14	0.41	0.66	0.74	0.34	-0.14
Dibenz[al]pyrene	0.60	0.72	0.27	0.04	0.45	0.60	0.72	0.27	0.04
Dibenz[ae]pyrene	0.54	0.67	0.30	-0.05	0.34	0.54	0.67	0.30	-0.05
Naphtho[2,3-e]pyrene	0.53	0.63	0.41	-0.22	0.10	0.53	0.63	0.41	-0.22
Retene	0.66	0.85	0.16	-0.10	0.18	0.66	0.85	0.16	-0.10
4-Methylpyrene	0.78	0.83	0.45	-0.05	0.41	0.78	0.83	0.45	-0.05
2-Methylpyrene	0.76	0.84	0.41	-0.03	0.42	0.76	0.84	0.41	-0.03
1-Methylpyrene	0.76	0.84	0.40	-0.03	0.41	0.76	0.84	0.40	-0.03
Xanthone	0.71	0.81	0.49	-0.15	0.30	0.71	0.81	0.49	-0.15
9,10-Anthracenedione	0.79	0.76	0.51	-0.04	0.38	0.79	0.76	0.51	-0.04
Cyclopenta(def)phenanthrenone	0.62	0.67	0.51	-0.18	0.29	0.62	0.67	0.51	-0.18
1,8-Naphthalic anhydride	0.69	0.56	0.44	0.01	0.31	0.69	0.56	0.44	0.01
11H-Benzo[a]fluoren-11-one	0.70	0.85	0.45	-0.10	0.39	0.70	0.85	0.45	-0.10
7H-Benzo[c]fluorene-7-one	0.73	0.86	0.45	-0.12	0.37	0.73	0.86	0.45	-0.12
11H-Benzo[b]fluoren-11-one	0.74	0.85	0.46	-0.12	0.38	0.74	0.85	0.46	-0.12
7H-Benzo[de]anthracen-7-one	0.73	0.83	0.45	-0.14	0.41	0.73	0.83	0.45	-0.14
Benz[a]anthracene-7,12-dione	0.75	0.60	0.42	-0.18	0.43	0.75	0.60	0.42	-0.18
Nicotine	0.38	0.60	-0.05	0.17	0.45	0.38	0.60	-0.05	0.17
Galactosan	0.53	0.74	0.44	-0.03	0.34	0.53	0.74	0.44	-0.03
Mannosan	0.50	0.76	0.36	-0.03	0.32	0.50	0.76	0.36	-0.03
Levoglucosan	0.61	0.75	0.65	-0.16	0.16	0.61	0.75	0.65	-0.16
Dehydroabietic acid, methyl ester	0.30	0.43	0.16	-0.04	0.10	0.30	0.43	0.16	-0.04
Dehydroabietic acid	0.44	0.67	0.11	-0.05	0.29	0.44	0.67	0.11	-0.05
29ab	0.52	0.55	0.44	0.03	0.46	0.52	0.55	0.44	0.03
30ab	0.50	0.48	0.45	0.05	0.47	0.50	0.48	0.45	0.05
31abS	0.54	0.42	0.59	-0.04	0.41	0.54	0.42	0.59	-0.04
31abR	0.52	0.40	0.63	-0.03	0.39	0.52	0.40	0.63	-0.03
32abS	0.62	0.46	0.66	-0.07	0.47	0.62	0.46	0.66	-0.07
32abR	0.64	0.51	0.71	-0.14	0.43	0.64	0.51	0.71	-0.14
33abS	0.54	0.28	0.73	-0.14	0.15	0.54	0.28	0.73	-0.14
33abR	0.54	0.34	0.77	-0.20	0.19	0.54	0.34	0.77	-0.20
Eicosane	0.62	0.73	0.32	0.05	0.40	0.62	0.73	0.32	0.05
Heneicosane	0.55	0.76	0.33	0.01	0.33	0.55	0.76	0.33	0.01
Docosane	0.58	0.78	0.39	0.00	0.33	0.58	0.78	0.39	0.00
Tricosane	0.64	0.78	0.49	-0.05	0.32	0.64	0.78	0.49	-0.05

Tetracosane	0.57	0.70	0.46	-0.12	0.29	0.57	0.70	0.46	-0.12
Pentacosane	0.43	0.51	0.40	-0.15	0.19	0.43	0.51	0.40	-0.15
Hexacosane	0.31	0.38	0.31	-0.15	0.13	0.31	0.38	0.31	-0.15
Heptacosane	0.30	0.35	0.33	-0.15	0.13	0.30	0.35	0.33	-0.15
Octacosane	0.27	0.32	0.29	-0.16	0.11	0.27	0.32	0.29	-0.16
Nonacosane	0.44	0.42	0.49	-0.23	0.14	0.44	0.42	0.49	-0.23
Triacontane	0.23	0.29	0.22	-0.13	0.10	0.23	0.29	0.22	-0.13
Hentriacontane	0.48	0.49	0.46	-0.17	0.21	0.48	0.49	0.46	-0.17
Dotriacontane	0.35	0.42	0.31	-0.11	0.19	0.35	0.42	0.31	-0.11
Tritriacontane	0.68	0.73	0.55	-0.12	0.34	0.68	0.73	0.55	-0.12
Tetratriacontane	0.65	0.76	0.39	-0.04	0.38	0.65	0.76	0.39	-0.04
Pentatriacontane	0.69	0.84	0.42	0.00	0.48	0.69	0.84	0.42	0.00
Hexatriacontane	0.63	0.82	0.27	-0.08	0.37	0.63	0.82	0.27	-0.08
Octatriacontane	0.65	0.88	0.19	-0.13	0.33	0.65	0.88	0.19	-0.13
Heptatriacontane	0.67	0.89	0.15	-0.17	0.43	0.67	0.89	0.15	-0.17
Nonatriacontane	0.64	0.86	0.18	-0.12	0.33	0.64	0.86	0.18	-0.12
Tetracontane	0.69	0.89	0.23	-0.14	0.35	0.69	0.89	0.23	-0.14

Supplementary Table 3. Average $DCFH_m$, DTT_m and AA_m values determined by multilinear regression (MLR) on volume-normalized measured assays (as response variable) and OA and elemental sources (as predictor variable). MLR was performed by providing 0 as lower bound (averaged over 100 bootstrap runs).

	$DCFH_m$ (average)	DTT_m (average)	AA_m (average)
CPOA	-	-	-
BBOA	-	-	1.10
COOA	0.07	0.11	-
UOOA	0.22	0.09	0.20
HOA	-	0.56	0.32
Factor 1a	$1.42*10^{-14}$	-	-
Factor 1b	-	-	-
Cu,Cd-rich	-	1.07	2.46
Pb, Sn rich	-	-	-
K^+-Na^+ rich	-	-	-
Factor 2a	-	-	0.05
Factor 2b	$2.09*10^{-11}$	-	$1.56*10^{-11}$
Factor 2c	2.02	22.07	-
Factor 2d	$4.14*10^{-13}$	0.09	0.09
Factor 2e	-	-	$7.1*10^{-12}$

Supplementary Table 4. Chemical composition and other auxiliary measurements conducted on the collected filters. A total of 300 filter samples (including field blanks) referred here as "All" were analyzed for EC-OC, WSOC, water soluble inorganic carbon, ions, and trace elements. Bulk and near-molecular level information on organic aerosols (OAs) was obtained using the offline technique developed at PSI[28,29]. A subset of 140 filters selected from four sites (CCSHAU, IITMD, IITD and MRIUF) were analyzed for targeted organic compounds. Another subset of 193 filters from CCSHAU, IITMD and IITD were analyzed for ^{14}C of TC and 44 filters out of 193 for ^{14}C of EC.

**Analytical method**	**Measured compounds**		**Filters measured**
Offline LToF-AMS	Bulk organics		All
Offline EESI-LToF-MS	Near-molecular level organics		All
Ion Chromatography	Ions (K^+, Na^+, NH_4^+, Cl^-, NO_3^-, SO_4^{2-})		All
Thermal optical transmittance using Sunset Lab Analyzer	Organic carbon - Elemental carbon (OC-EC) using EUSAAR 2 protocol (Cavalli et al., 2010; Bhowmik et al, 2021)		All
TOC-L analyzer - CO_2 quantification using a nondispersive infrared spectrophotometer; measuring CO_2 evolved after acidification (Bhowmik et al., 2021)	WSOC; water-soluble inorganic carbon (WSIC)		All
Inductive coupled plasma mass spectrometry (ICP-MS)	Trace elements (Li, Mg, As, Ca, Sc, Ti, V, Cr, Mn, Fe, Co, Ni, Cu, Se, Rb, Sr, Zr, Mo, Pd, Cd, ln, Sn, Sb, Cs, Ba, Ce, Pt, Tl and Pb); water-soluble Cu		All; 29 (across all sites)
	C4 dicarboxylic acid	Maleic acid	
		Succinic acid	
		Methylmalonic acid	
	C5 dicarboxylic acid	Citraconic acid	
		Glutaric acid	
		Methylsuccinic acid	
	C6 dicarboxylic acid	Adipic acid	
		Methylglutaric acid	

LC-MS (Borlaza et al., 2021)			140 samples [every 6th day sample from CCSHAU (33), IITD (36), IITMD (37) and MRIUF (34)]
	C7 dicarboxylic acid	Oxoheptanedioic acid	
	C8 dicarboxylic acid	Suberic acid	
		3-MBTCA acid	
		Phthalic acid	
	C9 dicarboxylic acid	Pinic acid	
		Azelaic acid	
	C10 dicarboxylic acid	Sébacic acid	
In-situ derivatization thermal desorption GC-ToF-MS (Orasche et al., 2011)	PAHs	Fluoranthene	140 samples [every 6th day sample from CCSHAU (33), IITD (36), IITMD (37) and MRIUF (34)]
		Acephenanthrylene	
		Pyrene	
		Benz[a]anthracene	
		Chrysene	
		sum_Benzo[b,k]fluoranthene	
		Benz[e]pyrene	
		Benz[a]pyrene	
		Perylene	
		Anthanthrene	
		Dibenz[ah]anthracene	
		Indeno[1,2,3-cd]pyrene	
		Picene	
		Benzo[ghi]perylene	
		Coronene	
		Naphtho[1,2-kb]fluoranthene	
		Dibenz[al]pyrene	
		Dibenz[ae]pyrene	
		Naphtho[2,3-e]pyrene	
		4-Methylpyrene	
		2-Methylpyrene	
		1-Methylpyrene	
	Oxy-PAHs	9,10-Anthracenedione	
		Cyclopenta(def)phenanthrenone	
		1,8-Naphthalic anhydride	
		1,8-Naphthalaldehydic acid	
		11H-Benzo[a]fluoren-11-one	
		7H-Benzo[c]fluorene-7-one	
		11H-Benzo[b]fluoren-11-one	
		7H-Benzo[de]anthracen-7-one	
	Anhydrous Sugar	Galactosan	
		Mannosan	
		Levoglucosan	
	Resin acids	Dehydroabietic acid, methyl ester	
		Dehydroabietic acid	
	Alkaloid	Cholesterol	
		á-Sitosterol	
		Nicotine	
	Hopanes	29ab	
		29ba	
		30ab	
		30ba	
		31abS	
		31abR	
		32abS	
		32abR	
		33abS	
		33abR	
	n-alkanes	Eicosane	
		Heneicosane	
		Docosane	
		Tricosane	
		Tetracosane	
		Pentacosane	
		Hexacosane	
		Heptacosane	
		Octacosane	
		Nonacosane	
	Higher n-alkanes	Triacontane	
		Hentriacontane	
		Dotriacontane	
		Tritriacontane	

		Tetratriacontane	
		Pentatriacontane	
		Hexatriacontane	
	Lignin pyrolysis products	Vanillic acid	
		Syringic acid	
Radiocarbon (^{14}C) measurements	^{14}C of TC and EC		[TC: All], [EC: 44 (Samples from CCSHAU, IITD and IITMD)]
Inductive Coupled Plasma Mass Spectrometry (ICP-MS)	Water soluble Cu^{2+}		31 (across all sites)

Supplementary Table 5. Variation of factor-specific recoveries (R_k, in percentage) and OM:OC ratio.

	Q_{25}	Q_{50}	Q_{75}	$(\frac{OM}{OC})_k$
CPOA	52.3	53.2	61.6	1.5
BBOA	75.3	99.8	100.0	1.7
COOA	92.3	95.5	98.5	2.5
UOOA	90.8	94.4	100.0	2.1
Factor 1a	80.1	93.9	93.2	2.1
Factor 1b	91.2	87.8	95.3	1.9

Supplementary Table 6. Fitting coefficients of relative (A) fossil and (B) non-fossil contributions of different factors determined by multilinear regression.

Multilinear regression on OC_f								
Factors	OC_{nf}				OC_f			
	Median	Q_{25}	Q_{50}	Q_{75}	Median	Q_{25}	Q_{50}	Q_{75}
CPOC	0.71	0.68	0.70	0.73	0.29	0.27	0.30	0.31
BBOC	0.98	1	1	1	0.01	-	-	-
COOC	0.81	0.81	0.82	0.82	0.18	0.17	0.18	0.19
UOOC	0.48	0.47	0.48	0.49	0.52	0.51	0.52	0.53
Multilinear regression on OC_{nf}								
CPOC	0.88	0.85	0.88	0.91	0.12	0.09	0.12	0.15
COOC	0.85	0.84	0.85	0.87	0.15	0.14	0.15	0.16
UOOC	0.56	0.55	0.56	0.57	0.44	0.43	0.44	0.45

Supplementary Table 7. AMS vs. EESI PMF correlation matrix. AMS and EESI PMF factor time series from the final solution were normalized to labelled $Na_2{}^{34}SO_4$ and $^{15}NO_3^-$ or $^{34}SO_4^{2-}$, respectively.

	$CPOA_{AMS}$	$BBOA_{AMS}$	$COOA_{AMS}$	$UOOA_{AMS}$	Factor $1a_{AMS}$	Factor $1b_{AMS}$
$CPOA_{EESI}$	**0.87**	0.65	0.43	-0.04	0.29	-0.16
$BBOA_{EESI}$	0.43	**0.73**	0.16	-0.06	-0.16	0.32
$COOA_{EESI}$	0.25	0.18	**0.81**	0.002	0.23	-0.10
$UOOA_{EESI}$	-0.10	0.003	-0.24	**0.42**	-0.24	0.25
Factor $1a_{EESI}$	-0.03	0.04	-0.23	0.08	-0.45	**0.62**
Factor $1b_{EESI}$	-0.10	-0.07	-0.17	-0.20	**0.42**	0.30

Supplementary Table 8. Correlation matrix of total $PM_{2.5}$ and its constituents and their sources with OP_v.

	All sites			CCSHAU			IITMD			IITD			MRIUF			IITK		
	$DCFH_v$	AA_v	DTT_v	$DCFH_v$	AA_v	DTT_v	$DCFH_v$	AA_v	DTT_v	$DCFH_v$	AA_v	DTT_v	$DCFH_v$	AA_v	DTT_v	$DCFH_v$	AA_v	DTT_v
AA_v	0.60**			0.72**			0.49**			0.42**			0.82**			0.82**		
DTT_v	0.43**	0.56**		0.64**	0.53**		0.18	0.57**		0.57**	0.64**		0.54**	0.64**		0.53**	0.71**	
$PM_{2.5}$	0.46**	0.54**	0.53**	0.12	0.49**	0.38**	0.43**	0.52**	0.35**	0.53**	0.50**	0.65**	0.57**	0.55**	0.73**	0.66**	0.69**	0.68**
AA_m	0.23	0.59**	0.02	0.18	0.56**	-0.15	0.44*	0.64**	0.17	-0.09	0.62**	0.04	-0.12	0.65**	0.21	0.40	0.37	-0.11
DTT_m	-0.07	0.06	0.14	0.10	0.23	0.01	0.08	0.16	0.49*	-0.18	0.06	0.15	-0.16	0.11	0.11	-0.21	-0.26	-0.23
$DCFH_m$	0.64**	0.20	-0.10	0.56**	0.20	-0.15	0.70**	0.07	-0.24	0.21	-0.16	-0.28	0.66*	-0.07	-0.04	0.67**	0.30	-0.04
OC	0.55**	0.68**	0.55**	0.31	0.65**	0.47	0.46	0.57	0.35	0.60**	0.65**	0.72**	0.47	0.65**	0.77**	0.70**	0.80**	0.65**
EC	0.31**	0.53**	0.35**	0.32*	0.44**	0.33*	0.34*	0.57**	0.55**	0.35*	0.80**	0.58**	0.12	0.65**	0.68**	0.53**	0.50**	0.41*
NO_3^-	0.31**	0.24**	0.30**	0.05	0.17	0.08	0.50**	0.44**	0.20	0.43*	0.13	0.35*	0.65**	0.30	0.51**	0.55**	0.45	0.57**
SO_4^{2-}	0.31**	0.27**	0.38**	0.05	0.20	0.27**	0.50**	0.41**	0.30*	0.37**	0.12	0.26	0.50**	0.36	0.58**	0.20	0.13	0.52
Cl^-	0.03	0.05	0.13	0.50**	0.57**	0.43**	-0.12	0.05	0.09	-0.12	-0.10	0.03	0.28	0.26	0.40	0.58**	0.67**	0.63**
CPOA	.04**	0.59**	0.39**	0.08	0.68**	0.35*	0.28*	0.40**	0.25	0.18	0.21	0.26	0.45**	0.60**	0.65**	0.66**	0.86**	0.56**
BBOA	0.47**	0.63**	0.38**	0.07	0.57**	0.23	0.25	0.34**	0.26*	0.39**	0.65**	0.54**	0.34**	0.67**	0.54*	0.72**	0.83**	0.56**
COOA	0.32**	0.32**	0.47**	0.05	0.40**	0.53**	0.29*	0.36**	0.15	0.40**	0.15	0.29*	0.32**	0.30*	0.53**	0.52**	0.50**	0.62**
UOOA	0.23**	0.07	0.02	0.36**	-0.04	-0.10	0.46**	0.25*	0.33*	-0.01	0.001	0.03	0.07	0.14	0.17	-0.30*	-0.50*	-0.30

**Cu-Cd rich**	0.01	0.32	0.19	0.46**	0.41**	0.36*	0.43**	**0.93****	0.44**	-0.04	0.25	0.38*	-0.08	0.29	0.13	0.05	0.26	0.23
**Pb-Sn-Sb rich**	0.04	0.22	0.16	0.43**	0.16	0.14	0.20	**0.60****	0.34	0.03	0.21	0.44**	0.13	0.40*	0.39*	0.31	0.12	0.12
**$K^{=}$-Na^{+} rich**	0.36*	0.30	0.32	0.06	0.38*	0.18	**0.56****	0.22	0.11	**0.65****	0.40**	0.44**	0.37*	0.50**	0.51**	**0.60****	0.44**	0.40**

*p value < 0.05, ** p value < 0.01. Pearson's r > 0.55 are bold.

Supplementary References

1. Bhowmik, H. S. et al.Temporal and spatial variability of carbonaceous species (EC; OC; WSOC and SOA) in PM2.5 aerosol over five sites of Indo-Gangetic Plain, *Atmos. Poll. Res.* **12**, 375-390 (2021).

2. Tripathi, N. et al. Characteristics of VOC composition at urban and suburban sites of New Delhi, India in winter, J. *Geophys. Res.-Atmos.* **127** (2022).

3. DeCarlo P.F. et al. Field deployable, high-resolution, time-of-flight aerosol mass spectrometer, *Anal. Chem.*, **78**, 8281–8289 (2006).

4. Lopez-Hilfiker, F. D. et al. An extractive electrospray ionization time-of-flight mass spectrometer (EESI-TOF) for online measurement of atmospheric aerosol particles, *Atmos. Meas. Tech.* **12**, 4867–4886 (2019).

5. Lee, C. P. et al. Online Aerosol Chemical Characterization by Extractive Electrospray Ionization–Ultrahigh-Resolution Mass Spectrometry (EESI-Orbitrap), *Environ. Sci. Technol.* **54**, 3871-3880 (2020).

6. Sueper, D. et al. A community software for quality control and analysis of data from the aerodyne time-of-flight aerosol mass spectrometers (ToF-AMS), *Annual Conference of the American Association for Aerosol Research,Reno, Nev (2007).*

7. Salazar, G., Zhang, Y. L., Agrios, K. & Szidat, S. Development of a method for fast and automatic radiocarbon measurement of aerosol samples by online coupling of an elemental analyzer with a MICADAS AMS, *Nucl. Instrum. Methods Phys. Res.* **361 (B),** 163-167 (2015).

8. Zhang, Y. L. et al. On the isolation of OC and EC and the optimal strategy of radiocarbon-based source apportionment of carbonaceous aerosols, *Atmos. Chem. Phys.* **12**, 10841–10856 (2012).

9. Szidat, S. et al. ^{14}C analysis and sample preparation at the new Bern Laboratory for the Analysis of Radiocarbon with AMS (LARA), *Radiocarbon* **56**, 561–566 (2014).

10. Agrios, K. et al. Online coupling of pure O_2 thermo-optical methods – ^{14}C AMS for source apportionment of carbonaceous aerosols study, *Nucl. Instrum. Meth. Phys. Res. B.* **361**, 288–293 (2015).

11. Stuiver, M. & Polach, H. A. Reporting of C-14 data – discussion, *Radiocarbon* **19**, 355–363 (1977).

12. Zhang, Y. L. et al. Fossil and nonfossil sources of organic and elemental carbon aerosols in the outflow from Northeast China, *Environ. Sci. Technol.* **50**, 6284–6292 (2016).

13. Zotter, P. et al. Radiocarbon analysis of elemental and organic carbon in Switzerland during winter-smog episodes from 2008 to 2012 – Part 1: Source apportionment and spatial variability, *Atmos. Chem. Phys.* **14**, 13551–13570 (2014).

14. Cavalli, F., Viana, M., Yttri, K. E., Genberg, J. & Putaud, J. -P. Toward a standardised thermal-optical protocol for measuring atmospheric organic and elemental carbon: the EUSAAR protocol, *Atmos. Meas. Tech.* **3**, 79–89 (2010).

15. Alleman, L. Y., Lamaison, L., Perdrix, E., Robache, A. & Galloo, J-C. PM_{10} metal concentrations and source identification using positive matrix factorization and wind sectoring in a French industrial zone, *Atm. Res.* **96**, 4, 612-625 (2010).

16. Borlaza, L. J. et al. Disparities in particulate matter (PM10) origins and oxidative potential at a city-scale (Grenoble, France) - Part I: Source apportionment at three neighbouring sites. *Atmos. Chem. Phys.* **21**, 5415–5437 (2021)

17. Orasche, J., Schnelle-Kreis, J., Abbaszade, G. & Zimmermann, R. Technical Note: In-situ derivatization thermal desorption GC-TOFMS for direct analysis of particle-bound non-polar and polar organic species, *Atmos. Chem. Phys.* **11**, 8977–8993 (2011).

18. Calas, A. et al. Comparison between five acellular oxidative potential measurement assays performed with detailed chemistry on PM_{10} samples from the city of Chamonix (France), *Atmos. Chem. Phys.* **18**, 7863–7875 (2018).

19. Borlaza, L. J. et al. Disparities in particulate matter (PM_{10}) origins and oxidative potential at a city scale (Grenoble, France) – Part 2: Sources of PM_{10} oxidative potential using multiple linear regression analysis and the predictive applicability of multilayer perceptron neural network analysis, *Atmos. Chem. Phys.* **21**, 9719–9739 (2021).

20. Campbell, S. J. et al. Atmospheric conditions and composition that influence $PM_{2.5}$ oxidative potential in Beijing, China, *Atmos. Chem. Phys.* **21**, 5549–5573 (2021).

21. Murrant C. L. & Reid, M. B. Detection of reactive oxygen and reactive nitrogen species in skeletal muscle, *Microsc. Res. Tech.* **55**, 236-248, 2001.

22. Polissar, A. V., Hopke, P. K., Paatero, P., Malm, W. C. & Sisler, J. F. Atmospheric aerosol over Alaska: 2. Elemental composition and sources. *J. Geophys. Res. Atmos.* **103**, 19045–19057 (1998)

23. Reff, A., Eberly, S. I. & Bhave, P. V. Receptor modeling of ambient particulate matter data using positive matrix factorization: review of existing methods, *J. Air Waste Manage. Assoc.* **57**, 146–154 (2007).

24. Ooki, A., Uematsu, M., Miura, K. & Nakae, S. Sources of sodium in atmospheric fine particles, *J. Atmos. Environ.,* **36(27)**, 4367-4374 (2002).

25. Rai, P. et al. Real-time measurement and source apportionment of elements in Delhi's atmosphere. *Sci. of Tot. Environ.* **742**, 140332, 2020.

26. Gao, D., Godri Pollitt, K. J., Mulholland, J. A., Russell, A. G. & Weber, R. J. Characterization and comparison of $PM_{2.5}$ oxidative potential assessed by two acellular assays, *Atmos. Chem. Phys.* **20**, 5197–5210, 2020.

27. Cheng, Y. et al. Biomass burning contribution to Beijing aerosol, *Atmos. Chem. Phys.* **13**, 7765–7781 (2013).

28. Däellenbach, K. R. et al. Characterization and source apportionment of organic aerosol using offline aerosol mass spectrometry, *Atmos. Meas. Tech.* **9**, 23–39 (2016).

29. Qi, L. et al. Organic aerosol source apportionment in Zurich using an extractive electrospray ionization time-of-flight mass spectrometer (EESI-TOF-MS) – Part 2: Biomass burning influences in winter, *Atmos. Chem. Phys.,* **19**, 8037–8062 (2019).
